# Single-Cell RNA-Sequencing and Optical Electrophysiology of Human Induced Pluripotent Stem Cell-Derived Cardiomyocytes Reveal Discordance Between Cardiac Subtype-Associated Gene Expression Patterns and Electrophysiological Phenotypes

**DOI:** 10.1089/scd.2019.0030

**Published:** 2019-05-13

**Authors:** Sherri M. Biendarra-Tiegs, Xing Li, Dan Ye, Emma B. Brandt, Michael J. Ackerman, Timothy J. Nelson

**Affiliations:** ^1^ Department of Molecular Pharmacology and Experimental Therapeutics, Mayo Clinic, Rochester, Minnesota.; ^2^ Center for Regenerative Medicine, Mayo Clinic, Rochester, Minnesota.; ^3^ Division of Biomedical Statistics and Informatics, Department of Health Sciences Research, Mayo Clinic, Rochester, Minnesota.; ^4^ Windland Smith Rice Sudden Death Genomics Laboratory, Department of Molecular Pharmacology and Experimental Therapeutics, Mayo Clinic, Rochester, Minnesota.; ^5^ Division of Heart Rhythm Services, Department of Cardiovascular Medicine, Mayo Clinic, Rochester, Minnesota.; ^6^ Division of Pediatric Cardiology, Department of Pediatric and Adolescent Medicine, Mayo Clinic, Rochester, Minnesota.; ^7^ Division of General Internal Medicine, Department of Internal Medicine, Mayo Clinic, Rochester, Minnesota.

**Keywords:** induced pluripotent stem cells, cardiomyocyte, differentiation, gene expression, electrophysiology

## Abstract

The ability to accurately phenotype cells differentiated from human induced pluripotent stem cells (hiPSCs) is essential for their application in modeling developmental and disease processes, yet also poses a particular challenge without the context of anatomical location. Our specific objective was to determine if single-cell gene expression was sufficient to predict the electrophysiology of iPSC-derived cardiac lineages, to evaluate the concordance between molecular and functional surrogate markers. To this end, we used the genetically encoded voltage indicator ArcLight to profile hundreds of hiPSC-derived cardiomyocytes (hiPSC-CMs), thus identifying patterns of electrophysiological maturation and increased prevalence of cells with atrial-like action potentials (APs) between days 11 and 42 of differentiation. To profile expression patterns of cardiomyocyte subtype-associated genes, single-cell RNA-seq was performed at days 12 and 40 after the populations were fully characterized with the high-throughput ArcLight platform. Although we could detect global gene expression changes supporting progressive differentiation, individual cellular expression patterns alone were not able to delineate the individual cardiomyocytes into atrial, ventricular, or nodal subtypes as functionally documented by electrophysiology measurements. Furthermore, our efforts to understand the distinct electrophysiological properties associated with day 12 versus day 40 hiPSC-CMs revealed that ion channel regulators *SLMAP*, *FGF12*, and *FHL1* were the most significantly increased genes at day 40, categorized by electrophysiology-related gene functions. Notably, *FHL1* knockdown during differentiation was sufficient to significantly modulate APs toward ventricular-like electrophysiology. Thus, our results establish the inability of subtype-associated gene expression patterns to specifically categorize hiPSC-derived cells according to their functional electrophysiology, and yet, altered *FHL1* expression is able to redirect electrophysiological maturation of these developing cells. Therefore, noncanonical gene expression patterns of cardiac maturation may be sufficient to direct functional maturation of cardiomyocytes, with canonical gene expression patterns being insufficient to temporally define cardiac subtypes of in vitro differentiation.

## Introduction

Despite widespread use of human induced pluripotent stem cells (hiPSCs), questions remain regarding to what degree cells differentiated from hiPSCs in vitro appropriately mimic their in vivo counterparts. Human induced pluripotent stem cell-derived cardiomyocytes (hiPSC-CMs) provide an excellent example, since the process of heart development involves the differentiation of distinct ventricular, atrial, and nodal subtypes of cardiomyocytes with unique phenotypes, including in both gene expression and functional measures such as electrophysiology [1–6].

Similar to what takes place during heart development, cardiac differentiation of pluripotent stem cells produces a diverse population of cardiomyocytes reported to exhibit ventricular-, atrial-, and nodal-like features [7,8]. This is of great importance for the application of these cells; ideally, the type of cardiomyocyte should suit its ultimate purpose. For example, it would be most relevant to study the mechanisms of an atrial-specific disease such as atrial fibrillation in atrial-like hiPSC-CMs or regenerate an injured ventricle using ventricular-like hiPSC-CMs. However, imperfect markers and inconsistent approaches lead to conflicting views of what constitutes a defining subtype characteristic, which thus hinders these downstream applications.

It has been postulated and disputed in published reports that distinct in vitro subpopulations of ventricular-, atrial-, and nodal-like hiPSC-CMs can be determined from their action potential (AP) waveforms as the gold standard [9,10]. This benchmark of cellular phenotyping is complicated by the in vitro conditions of hiPSC-CMs that only partially recapitulate the native conditions when comparing with cardiomyocytes found in homeostatic physiological systems. With increased sensitivity of the molecular readouts, canonical markers thought once to be tissue specific are now recognized to have a broader expression, a common example in the cardiac field being the atrial isoform of MLC2, which is expressed in both atria and ventricles during development [11,12].

Therefore, the inherent heterogeneity of hiPSC-CM differentiation processes still poses unmet challenges in their application to disease modeling, cell-based cardiac therapies, and chamber-specific drug toxicity testing [7,13]. Herein, we sought to determine the relationship between both molecular and functional properties as hiPSCs respond to a controlled differentiation protocol with the hope to reveal in vitro determinants of tissue specificity. There have been indications with highly sensitive readouts that the correlation between these cellular subtyping approaches may not be as clear cut as often assumed, exacerbated in the context of in vitro differentiation [9,14,15].

We wanted to further examine this paradigm, and chose to utilize hiPSC-CMs as our model system due to the reported presence of distinct subtypes of these cells, with both gene expression and electrophysiological markers commonly used to identify them. We were thus able to leverage the genetically encoded voltage indicator ArcLight and single-cell RNA-sequencing (scRNA-seq) to carefully profile the inherent molecular and functional complexity of the hiPSC-CM system and examine whether gene expression was predictive of electrophysiological phenotypes. Ultimately, this study was able to document the inability of canonical gene expression patterns to inform cardiomyocyte subpopulation classification in vitro, while identifying noncanonical molecular modulators of hiPSC-CM electrophysiology.

## Materials and Methods

A detailed description of all experimental procedures can be found in the Supplementary Data.

### ArcLight imaging and analysis

All ArcLight data acquisition was performed in a live-cell incubation chamber with 5% CO_2_ at 37°C. AP recordings were recorded from spontaneously beating cells using line-scan mode of a Zeiss 5 Live laser confocal microscope at 40 × magnification and 500 frames per second. An FITC filter set was used for measurements of ArcLight fluorescence. A custom MATLAB program (MathWorks) was written to analyze AP parameters from the recorded data.

### scRNA-seq and data analysis

The Fluidigm C1 system (middle-sized chip, 10–17 μm) was used to sort to individual cells from a single-cell suspension of hiPSC-CMs. Libraries were only prepared for capture sites containing a single intact cell. Paired-end sequencing was performed using an Illumina HiSeq 2500. In total, 42 cells on day 12 and 43 cells on day 40 were sequenced, similar in scope to other scRNA-seq studies, including another recent analysis of hiPSC-CMs [16–19].

Sequencing reads were mapped using standard messenger RNA (mRNA) sequencing workflow MAP-Rseq to generate read count matrix [20]. Differential analyses were performed using DESeq R software package [21]. Differentially expressed genes (DEGs) were selected based on *P* values <0.05 after false discovery rate control and log2-fold change >2.0. Enriched pathways on DEGs were selected by *P* values calculated by a Fisher test. Cells in subcluster cardiomyocyte analyses were selected based on cardiac marker expression and unsupervised hierarchical clustering.

## Results

### Differentiation and characterization of hiPSC-CMs

All hiPSCs were reprogrammed from dermal fibroblasts isolated from healthy individuals and differentiated to cardiomyocytes using a monolayer-based directed differentiation protocol. Standard quantitative reverse transcription-polymerase chain reaction (qRT-PCR) analysis of day 0 (day of initiation) through day 20 (D20) of differentiation showed temporal progression through pluripotency, precardiac and cardiac progenitor, and finally, cardiac gene expression (Supplementary Table S1). The latter included expression of quintessential ion channel genes as well as established atrial- and ventricular-associated genes. Several genes, such as the ventricular myosin gene *MYL2*, demonstrated increased expression over time, as expected within a progressive maturation process (Supplementary Fig. S1).

**FIG. 1.**
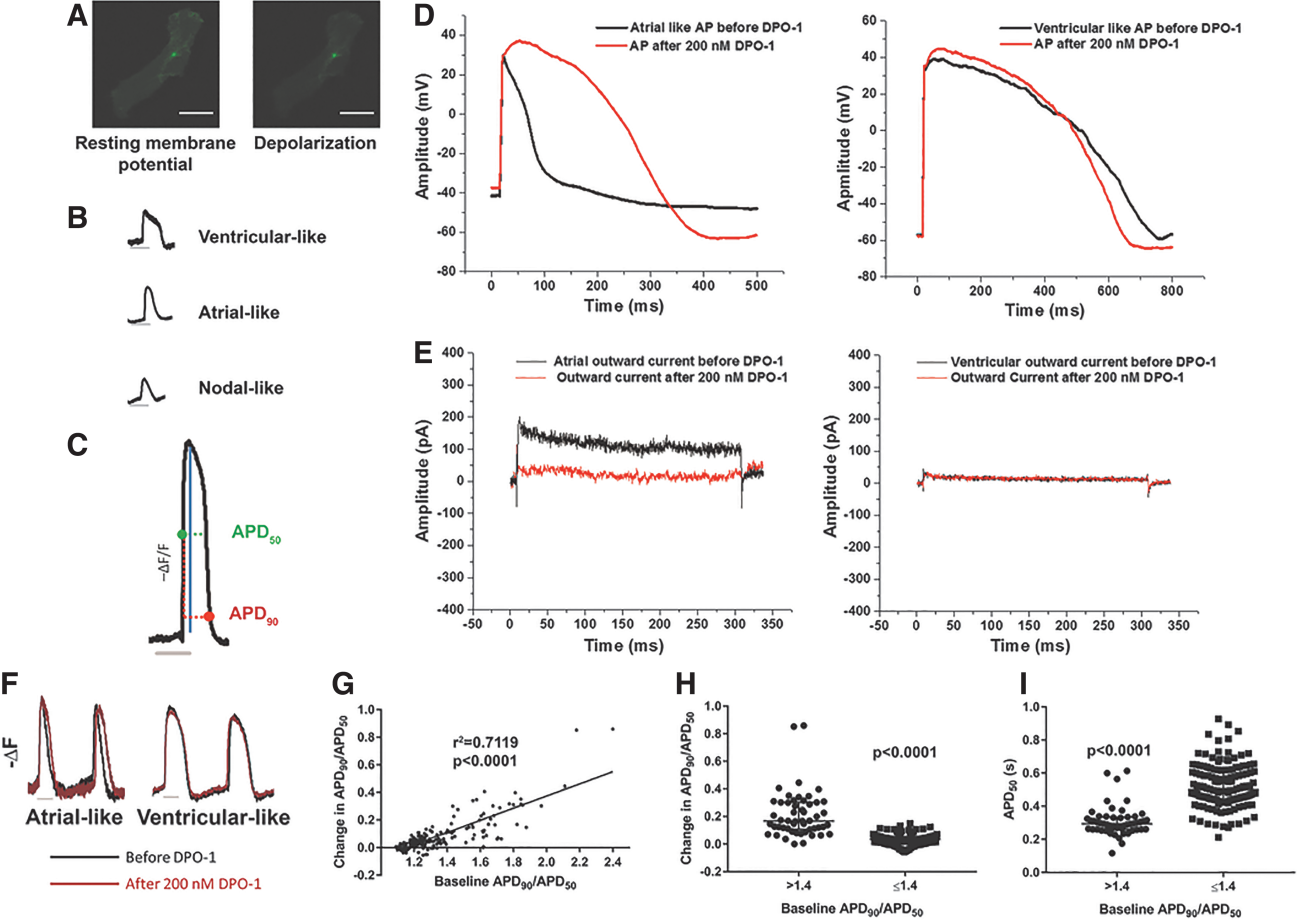
ArcLight demonstrates the ability to detect AP morphologies consistent with current density of I_Kur_. **(A)** ArcLight-expressing cells demonstrate decreased fluorescence with depolarization. Scale bar represents 20 μm. **(B)** Optical APs include those that resemble ventricular-like (D41), atrial-like (D41), and nodal-like (D42) morphologies. **(C)** Filtered optical tracings of negative change in fluorescence over fluorescence (−ΔF/F) allow analysis of AP properties, including APD_50_ (*horizontal green dotted line*), APD_90_ (*horizontal red dotted line*), amplitude (*blue line*), and V_max_ (not shown). **(D)** Representative traces for AP recordings before and after treatment with 200 nM DPO-1 using current clamp mode at a constant rate of 1 Hz through 5 ms depolarizing current injections of 150–300 pA. **(E)** Representative whole-cell outward currents before and after 200 nM DPO-1 elicited by depolarization of 300 ms duration to +40 mV from a holding potential of −50 mV. **(F)** Representative inverted fluorescence traces for optical APs before (*black*) and after (*red*) 200 nM DPO-1. **(G)** Change in APD_90_/APD_50_ (baseline − treatment) following DPO-1 administration, versus baseline APD_90_/APD_50_. Cells *n* = 190 from three to four differentiations each of three clones. Spearman correlation coefficient is shown. **(H)** Cells with baseline APD_90_/APD_50_ >1.4 adopt more ventricular-like AP morphology (larger change in APD_90_/APD_50_) following treatment with DPO-1. **(I)** Cells with baseline APD_90_/APD_50_ >1.4 exhibit shorter APD_50_. Cells for **(D–I)** are between D30 and D40 of differentiation. Trace bars represent 500 ms. All data are reported as median ± interquartile range. *P* values calculated via Mann–Whitney U test. AP, action potential; APD_50_, action potential duration at 50% repolarization; APD_90_, action potential duration at 90% repolarization; D, day; I_Kur_, ultrarapid delayed rectifier potassium current; V_max_, maximum upstroke velocity.

Following the formation of beating cardiomyocytes, which typically occurred around day 8 of directed differentiation, the ArcLight transgene was introduced to the hiPSC-CMs by lentiviral transduction to optically evaluate electrophysiology. Notably, green fluorescence in hiPSC-CMs expressing ArcLight was brighter at the resting potential and subsequently decreased with membrane depolarization, producing an optical AP. This was apparent in cell monolayers (Supplementary Fig. S2A), as well as in hiPSC-CMs, which were dissociated and more sparsely plated (Fig. 1A). We observed that these optical APs were temporally consistent with intracellular calcium flux (Supplementary Fig. S2B). APs included those that resembled ventricular, atrial, and nodal morphologies, consistent with previous reports (Fig. 1B).

We developed an analysis scheme to quantify several parameters of interest: AP amplitude, maximum upstroke velocity (V_max_), action potential duration at 50% or 90% repolarization (APD_50_, APD_90_), and interval between APs (Fig. 1C). Because ArcLight allows measurement of relative fluorescent signals rather than absolute membrane potentials, we could not measure maximum diastolic potential. Of particular note, ratios such as APD_90_/APD_50_ have previously been used to characterize hiPSC-CM subtype via patch clamp, with putative ventricular-like cells demonstrating a lower ratio, atrial-like cells demonstrating a higher ratio, and nodal-like cells at an intermediate value [11].

To validate this approach to evaluating electrophysiological properties of hiPSC-CMs, we confirmed that we could detect response to several prototypic drugs, including decreased AP interval and shortened AP duration with norepinephrine (Supplementary Fig. S2C), increased APD_90_/APD_50_ with hERG inhibitor E-4031 (Supplementary Fig. S2D), and shortened APD_50_ with L-type calcium channel inhibitor nifedipine (Supplementary Fig. S2E).

### Identification and quantification of atrial-like APs with ArcLight

Examination of AP profiles is one of the most common approaches to categorizing hiPSC-CMs into cardiomyocyte subtypes, and so, we first sought to validate a classification methodology that was both quantitative and calibrated to a subtype-specific ion current. We particularly wanted to be able to differentiate between ventricular- and atrial-like APs, which reportedly constitute the majority of those displayed by iPSC-CMs. The approach we settled on involved selectively inhibiting the atrial-enriched K_v_1.5 potassium channel and I_Kur_ (ultrarapid delayed rectifier potassium current) via the compound DPO-1.

We first verified the activity of this inhibitor via patch clamping (Supplementary Table S2). As expected, cells that qualitatively exhibited an atrial-like AP at baseline clearly responded to DPO-1 treatment by adopting a more ventricular-like AP morphology. Conversely, cells with more ventricular-like APs before treatment remained unaffected (Fig. 1D). Likewise, outward current was only reduced in the cells with atrial-like APs (Fig. 1E).

ArcLight was subsequently utilized to obtain a larger sample size and determine quantitative parameters by which to classify APs into atrial- or ventricular-like DPO-1 responders or nonresponders, respectively. We initially performed ArcLight analysis on the same differentiations as were analyzed by patch clamp (Supplementary Table S3). As originally observed via patch clamp, cells exhibiting a more qualitatively atrial-like AP signature and a larger APD_90_/APD_50_ ratio exhibited a pronounced response to DPO-1 treatment (Fig. 1F, G). The decreased APD_90_/APD_50_ ratio that we observed with DPO-1 treatment was distinctly different than the effect we had previously seen with I_Kr_ inhibition via E-4031 (Supplementary Fig. S2D).

We identified an APD_90_/APD_50_ ratio of 1.4 as being able to separate cells with a change in APD_90_/APD_50_ of at least 0.1 with >90% specificity and >80% sensitivity, and a change of at least 0.2 with >85% specificity and 100% sensitivity. This value is consistent with a parameter previously used to define ventricular and nonventricular APs via patch clamp, with the ventricular APs having lower APD_90_/APD_50_ values [11]. When we categorized the analyzed cells according to this value, we noted that cells with APD_90_/APD_50_ > 1.4 had a more profound change in APD_90_/APD_50_ with DPO-1 treatment compared with cells with lower APD_90_/APD_50_ (Fig. 1H). Likewise, cells with APD_90_/APD_50_ > 1.4 had shorter APD_50_ at baseline, which is consistent with atrial-like cardiomyocytes (Fig. 1I).

We also confirmed that the majority (78%) of cells produced from a retinoic acid-directed protocol, an established method for promoting an atrial-like phenotype in pluripotent stem cell-derived cardiomyocytes [13,22], had an APD_90_/APD_50_ ratio of >1.4 according to ArcLight analysis (Supplementary Fig. S2F).

Overall, we were able to determine the equivalency of patch clamp and ArcLight for phenotyping hiPSC-CMs, as both methodologies identified atrial-like DPO-1 responders, which had lower APD_50_ values and higher APD_90_/APD_50_ values at baseline compared with ventricular-like DPO-1 nonresponders (Supplementary Tables S2 and S3). We found that the majority of cells analyzed before DPO-1 treatment demonstrated ventricular-like APs according to both patch clamp and ArcLight evaluations of the same cell lines. In addition to being an appropriate surrogate for hiPSC-CM phenotyping via patch clamp, ArcLight facilitated the evaluation of nearly 200 individual cells.

We further attempted to characterize nodal cells, as distinguished by abolishment of automaticity in response to funny current (I_f_) inhibitor ivabradine [23]. As expected, only a small proportion of the cells responded (9 of 112, 8%). However, we could not determine any common distinct characteristics in the AP profiles of those cells (Supplementary Fig. S2G). Henceforth, we relied on published descriptions for nodal-like AP properties [6].

### Electrophysiological and gene expression shifts in a time course of hiPSC-CM differentiation

**FIG. 2.**
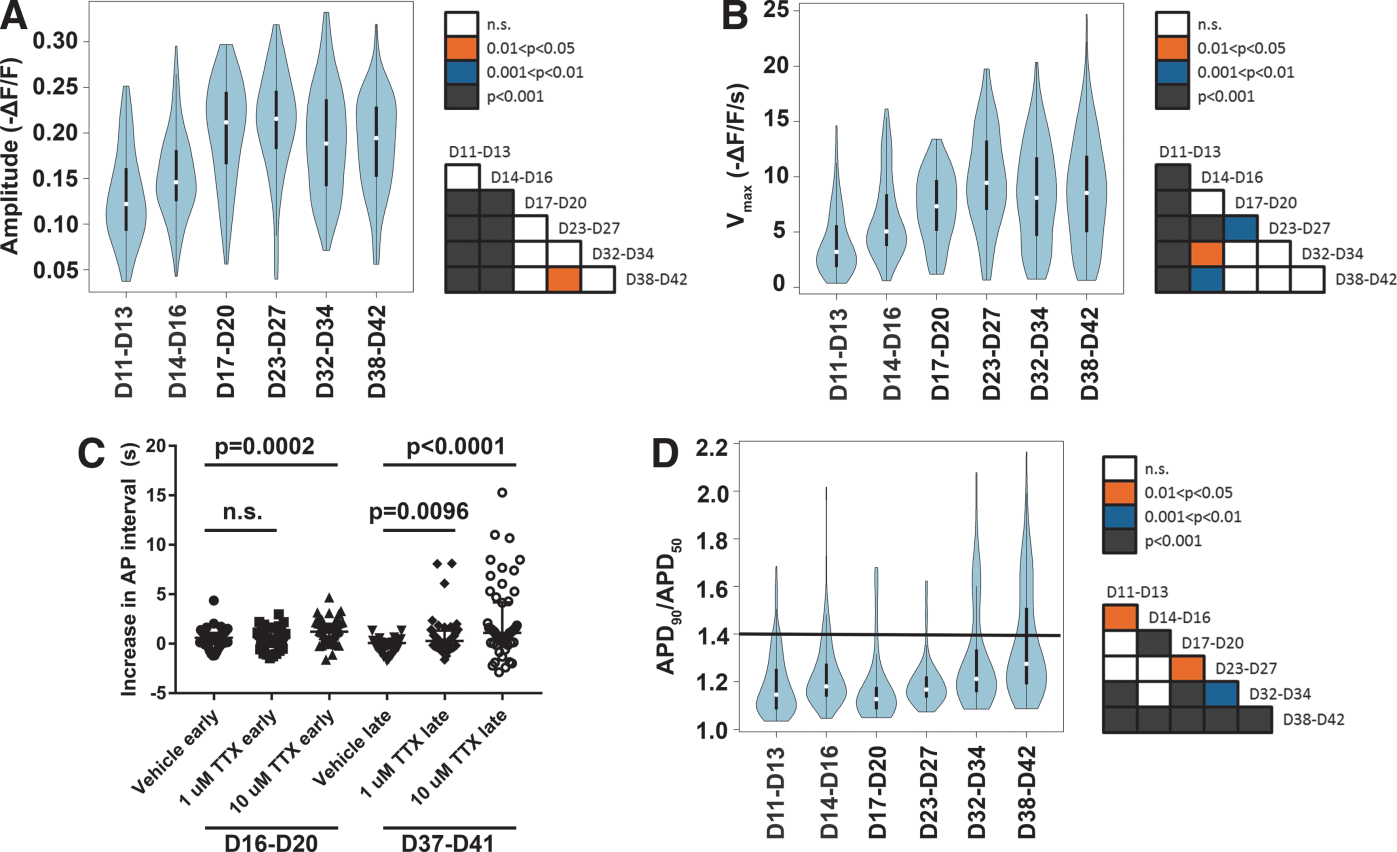
hiPSC-CMs demonstrate electrophysiological maturation and increased heterogeneity with extended time in culture. **(A)** Optical AP amplitudes are shifted toward larger values after day 16 of differentiation. **(B)** Optical AP V_max_ is shifted toward larger values after D16 of differentiation. **(C)** hiPSC-CMs between D37 and D40 of differentiation (late) respond to TTX with an increased interval between APs compared with the corresponding vehicle treatment. Cells between D16 and D20 of differentiation (early) also respond, but to a lesser degree. Cells were analyzed in six independent experiments from three different clones. Data are reported as median ± interquartile range. *P* values between vehicle and TTX treatments were calculated via a Mann–Whitney U test. **(D)** AP morphology, as described by APD_90_/APD_50_ (ratio of action potential durations at 90% and 50% repolarization), shows a shift in distribution with increased time in culture. The *horizontal line* marks APD_90_/APD_50_ ratio of 1.4. Data for **(A**, **B**, **D)** were collected from four to seven independent differentiations per time range. Three clones from unrelated individuals are represented. *White dots* within each violin indicate medians and *black rectangles* indicate interquartile range. Days 11–13 of differentiation: *n* = 121 cells; D11–D14: *n* = 123; D17–D20: *n* = 88; D23–D27: *n* = 121; D32–D34: *n* = 102; D38–D42: *n* = 166. Following statistical analysis via a Kruskal–Wallis test, pairwise comparisons were performed using Dunn's test with Bonferroni adjustment. Significance of pairwise comparisons is presented as *box color* in the corresponding matrix for each parameter. hiPSC-CMs, human-induced pluripotent stem cell-derived cardiomyocytes; TTX, tetrodotoxin.

Having established the utility of the ArcLight system and determined a classification system for APs, electrophysiological changes were mapped over the course of long-term culture of hiPSC-CMs (∼6 weeks of differentiation), a time frame during which a mixed population of cardiomyocyte subtypes has often been described [11,24–29]. Cardiomyocytes at D16 or earlier had a lower AP amplitude (Fig. 2A) and lower V_max_ (Fig. 2B) than those cells analyzed later in differentiation. Furthermore, cardiomyocytes between D37 and D41 were more sensitive to the sodium channel blocker tetrodotoxin than cardiomyocytes between D16 and D20 (Fig. 2C). Previously, reduced automaticity in response to tetrodotoxin treatment has been associated with a more advanced developmental stage [30].

Together, these three phenotypic changes are consistent with electrophysiological maturation over time in culture [31]. Notably, after approximately D30, a subpopulation of cardiomyocytes with APD_90_/APD_50_ values >1.4 emerged, which were seldom seen earlier in the differentiation process (Fig. 2D). Although we also noted changes in the interval between APs after D20 of differentiation, this did not appear to modulate AP duration as both APD_50_ and APD_90_ were largely unchanged across time points when examined individually (Supplementary Fig. S3).

We initially observed these temporal trends in AP amplitude, V_max_, and APD_90_/APD_50_ when using a defined medium (CDM3 [11]) for the cardiac differentiation protocol, but found they were also reproducible using B-27 medium (Supplementary Fig. S4). Thus, inherent differences in differentiation medium components did not appear to affect these phenotypes. The emergence of a population of cells with atrial-like electrophysiology along with the changes in maturation-related properties suggested that the hiPSC-CMs had matured to more defined cardiomyocyte subtypes following ∼1 month in culture, as determined from an electrophysiological standpoint.

**FIG. 3.**
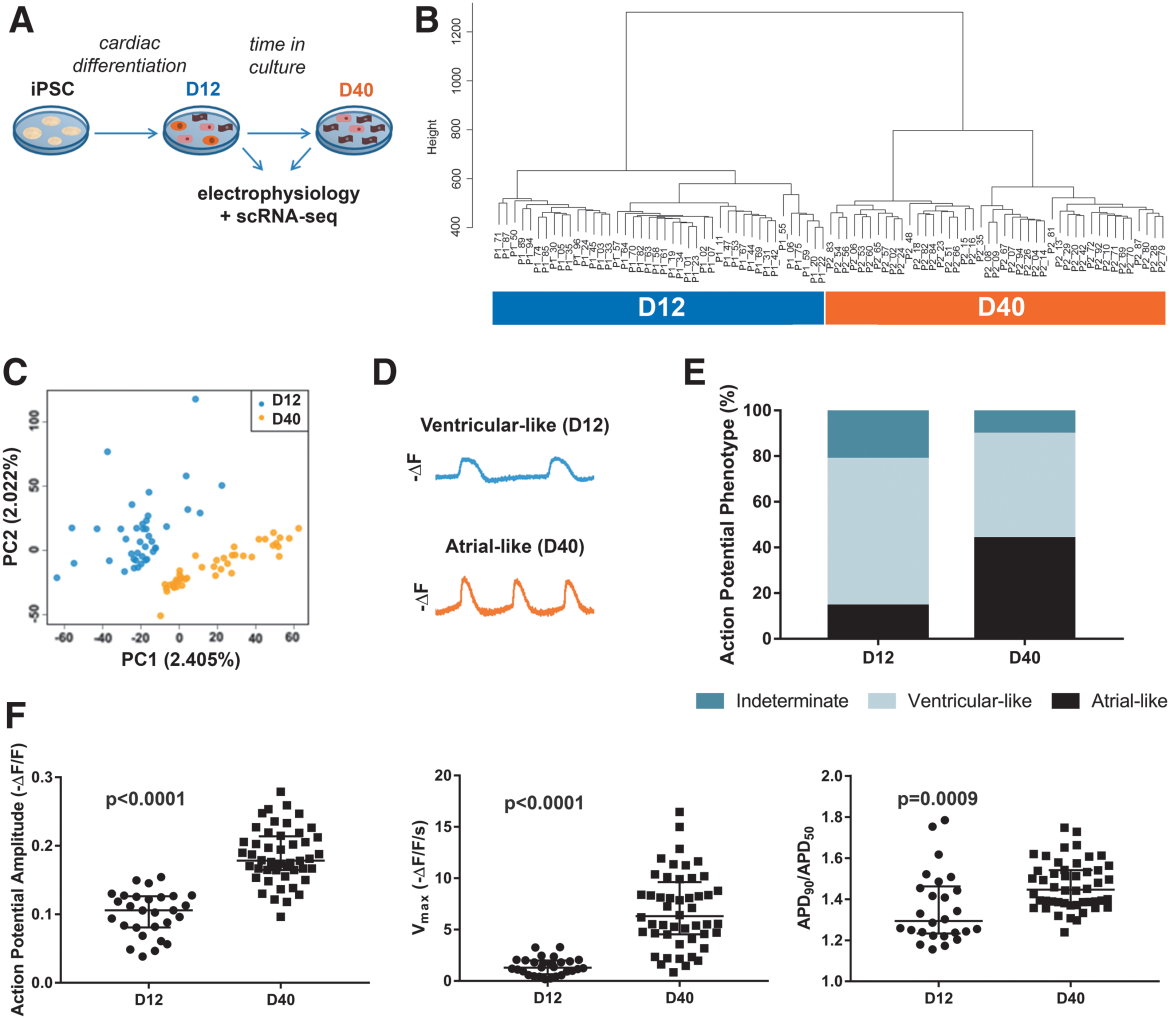
Single-cell RNA-seq analysis in hiPSC-derived cardiac differentiation clearly separates days 12 and 40 cells with distinct electrophysiological properties. **(A)** Schematic of experimental design. hiPSC-CMs were analyzed at days 12 and 40 of differentiation via single-cell RNA-seq and ArcLight. **(B)** Unsupervised hierarchical clustering on the entire transcriptomic profiles of all cells (D12: *n* = 42 cells; D40: *n* = 43 cells). **(C)** Principal component analysis compiled from the entire transcriptomic profiles of all cells. **(D)** Representative inverted fluorescent traces for ventricular-like morphology (from D12) and atrial-like morphology (from D40). **(E)** Percentage of total cells at D12 or D40 with each AP morphology classification. “Indeterminate” cells had either insufficiently mature APs or demonstrated a mixture of atrial- and ventricular-like APs. **(F)** AP amplitude (*left*), V_max_ (*middle*), and APD_90_/APD_50_ (*right*) evaluated from D12 and D40 populations before submission for sequencing. *P* values were calculated by either a Student's *t*-test or Mann–Whitney U test. Data are reported as median ± interquartile range. hiPSC, human induced pluripotent stem cell.

Next, we wanted to evaluate whether gene expression patterns would be predictive of the increased preponderance of cells with an atrial-like electrophysiology over time in culture. To evaluate changes in CM subtype marker and electrophysiology-related gene expression at high granularity and in a manner conducive to subgroup analysis, we performed both ArcLight analysis and scRNA-seq at day 12 (D12) and day 40 (D40) of differentiation (Fig. 3A). Our scRNA-seq data revealed dramatic transcriptome differences between D12 and D40 (Fig. 3B, C). Unsupervised hierarchical clustering (Fig. 3B) and principal component analysis (Fig. 3C) of expression data (including all genes in all sequenced cells) demonstrated that the selected time points segregated into two easily distinguishable groups exhibiting distinct transcriptional phenotypes.

From an electrophysiological standpoint, cells at D12 primarily displayed a ventricular-like AP morphology. At D40, the cells remained primarily ventricular like in their electrophysiology, but there was a more even distribution of cells with atrial-like and ventricular-like APs (Fig. 3D, E). A small subset of samples, particularly in the less electrophysiologically mature D12 population, was classified as “indeterminate” because they did not clearly resemble either atrial- or ventricular-like APs. APs that were distinctly nodal like were not identified. Overall, electrophysiological changes were consistent with what had previously been observed, namely increased AP amplitudes and V_max_ and a higher mean APD_90_/APD_50_ with longer time in culture (Fig. 3F).

### Heterogeneous expression of cardiomyocyte subtype-associated genes

Before evaluating cardiomyocyte subtype-associated markers, we wanted to confirm that our transcriptional data were consistent with cardiomyocyte development. We observed that each cell expressed transcripts for between ∼7,000 and ∼14,000 individual genes (Supplementary Fig. S5A). All cells had more than 1 million mapped reads, which has been described as sufficient for near maximal detection of expressed genes in scRNA-seq studies [32]. For more than 80% of the cells assayed, this value was between 5 and 10 million reads.

There was significantly higher expression of 732 genes at D12 and 271 genes at D40 (Supplementary Fig. S5B–F). Numerous transcription factors known to be involved in heart development were among the D12 DEGs, such as *NKX2.5* and *BMP2*. Functional enrichment analysis showed that the D12 DEGs could be categorized into functions consistent with cardiac development, including “atrial cardiac muscle tissue morphogenesis” and “ventricular septum development” (Supplementary Fig. S5G). qRT-PCR confirmed expression changes of several genes between D12 and D40, including known benchmark genes in heart development (Supplementary Fig. S5H, I). In addition, numerous D12 and D40 DEGs had previously been implicated specifically in early or later heart development, respectively [33,34] (Supplementary Fig. S6).

**FIG. 4.**
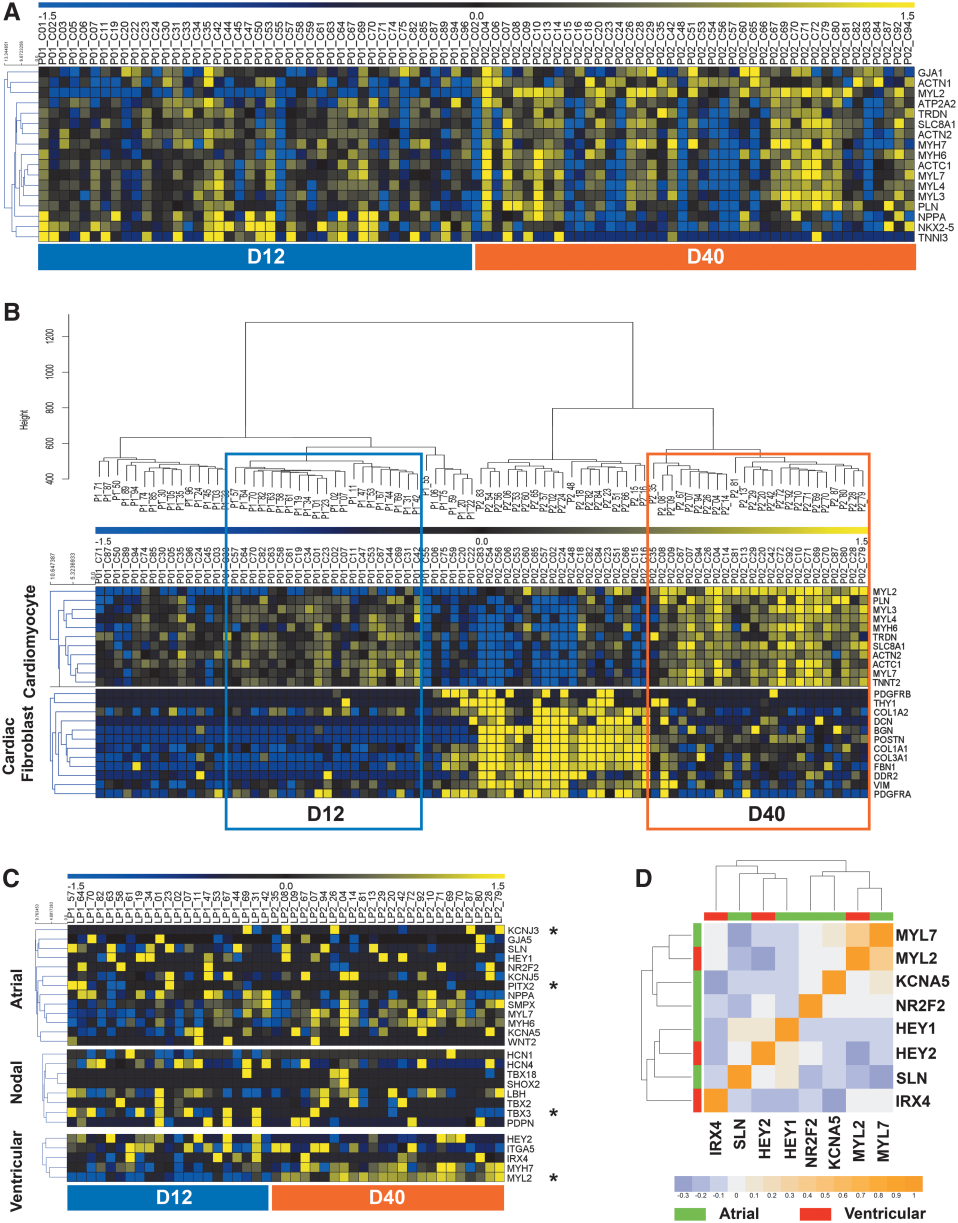
Single-cell RNA-seq profiles from hiPSC-CMs do not demonstrate distinct cardiomyocyte subtype commitment. **(A)** Heatmap of expression patterns for cardiomyocyte genes across all 85 cells on days 12 and 40 (each *column* representing one cell). *Blue* and *yellow* represent low and high expression, respectively. *MYL2* transcripts were detected in >25% of D12 and 100% of D40 cells, *NKX2.5* transcripts were detected in >75% of all cells, and all other transcripts were detected in every cell. **(B)** *Boxed* D12 and D40 cells were selected as cardiomyocytes for further analysis, according to unsupervised hierarchical clustering, robust expression of cardiomyocyte genes, and low expression of cardiac fibroblast genes. **(C)** Heatmap of expression patterns for atrial-, nodal-, and ventricular-associated genes in cardiomyocytes. Genes with significantly different expression (log_2_FC >2 and *P* < 0.05) between D12 and D40 are marked with an *asterisk*. **(D)** Heatmap representation of a correlation matrix for expression of atrial- and ventricular-associated genes, labeled in *green* (atrial) or *red* (ventricular). Positive and negative correlations are labeled as *orange* and *blue*, respectively.

We initially noted that many established cardiomyocyte genes had detectable expression in every cell analyzed (Fig. 4A). However, further analysis revealed heterogeneity in expression of these genes between individual cells. Unsupervised hierarchical clustering delineated subclusters within both the D12 and D40 populations (50% and 56% of the cells, respectively), which exhibited relatively higher expression levels of cardiomyocyte genes (Fig. 4B). We henceforth defined those subclusters as the cardiomyocyte populations, and focused specifically on those cells for further analyses. Many of the cells that did not fall into those subclusters had higher expression of cardiac fibroblast genes, which we further subcharacterized in the D40 population via immunofluorescence and additional analysis of the scRNA-seq data (Supplementary Fig. S7).

Differential gene expression analysis was repeated for D12 and D40 cardiomyocytes within the selected subclusters (Supplementary Fig. S8A). By specifically examining the cardiomyocytes within the mixed population, we were able to identify markers that were only detectable at either D12 or D40, and validated one of these markers (*LGALS3BP*) via immunofluorescence (Supplementary Fig. S8B, C).

Our transcriptome analysis confirmed DEGs that have previously been associated with early or late embryonic heart development and were more highly expressed at D12 or D40, respectively (Supplementary Fig. S8D). These D40 DEGs included *HOPX*, which has also been implicated as a cardiac maturation gene by two recent scRNA-seq studies of human pluripotent stem cell-derived cardiomyocytes [19,35]. Altogether, these results demonstrate that the D40 cells exhibited gene expression patterns associated with more mature cardiomyocytes compared with D12.

To classify the cells into cardiomyocyte subtypes according to their transcriptional signatures, we curated a panel of genes that have previously been used as markers of mature atrial-, ventricular-, and nodal-like cardiomyocytes, and examined expression of these genes in all hiPSC-CMs from D12 to D40 cultures. Possibly due to the lack of the exact microenvironment that exists for in vivo development processes, hiPSC-CMs show limited canonical expression patterns that correlated with a distinct subpopulation of a specific mature cardiac subtype.

Also, only a small set of subtype-associated genes exhibited a significantly different expression between the two populations, namely *MYL2*, *KCNJ3* (higher at D40), *TBX3*, and *PITX2* (higher at D12) (Fig. 4C). No clear pattern of gene expression could be discerned to definitively identify preferential development of a particular subtype in hiPSC-CMs over time, as these few DEGs were a mixture of ventricular, atrial, and nodal markers. We did not identify other D12 versus D40 cardiomyocyte DEGs that have been commonly used as markers of cardiomyocyte subtypes.

Examining coexpression patterns for a subset of common atrial or ventricular marker genes revealed only a few weak correlations between genes representing the same subtype (Fig. 4D). For example, *KCNA5* is of particular interest because it encodes the pore-forming subunit of atrial K_v_1.5. While it demonstrated a marginal positive correlation with the atrial myosin gene *MYL7*, it negatively correlated with expression of *SLN*. Overall, in contrast to the electrophysiological characterization, the gene expression data did not suggest that this culture system comprised distinct subgroups of primarily atrial-like and ventricular-like cells at D40 of in vitro differentiation.

### Changes in electrophysiology-related gene expression between D12 and D40 of differentiation

Since analysis of recognized cardiomyocyte subtype-associated genes did not provide clear insight into the electrophysiological phenotypes of these cells, we next looked at the expression patterns of ion channel and calcium-handling genes in the cardiomyocytes as a possible explanation for the time-dependent electrophysiological changes.

**FIG. 5.**
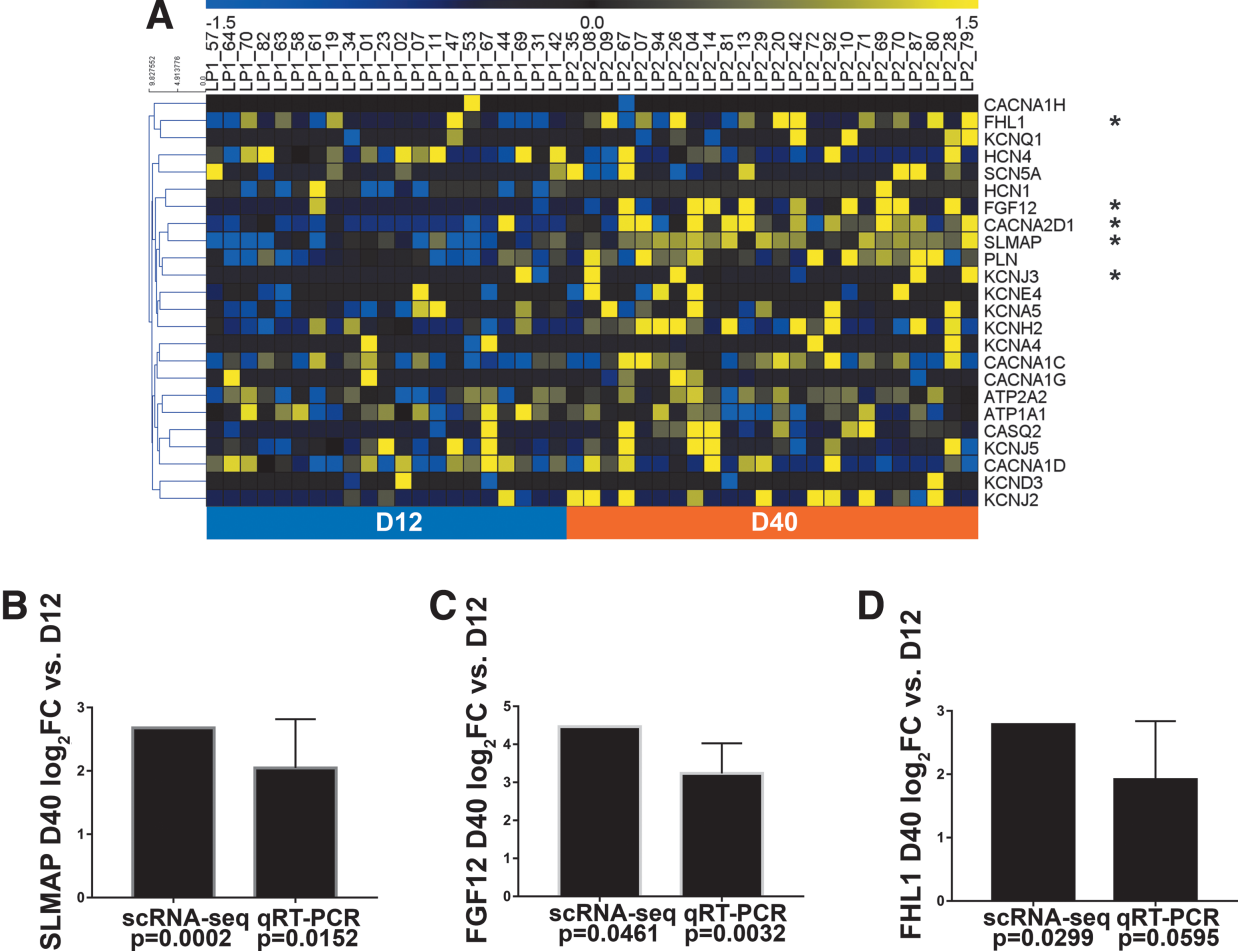
Several ion channel regulators exhibit increased expression with electrophysiological maturation of hiPSC-CMs. **(A)** Heatmap representation of expression patterns for representative electrophysiology- and calcium handling-associated genes in cardiomyocytes. Genes with significantly different expressions (log_2_FC >2 and *P* < 0.05) between days 12 and 40 are marked with an *asterisk*. **(B–D)** Relative expression (log_2_FC) of *SLMAP* **(B)**, *FGF12* **(C)**, or *FHL1* **(D)** D40 gene expression for both single-cell RNA-seq (cardiomyocytes only) and qRT-PCR, compared with D12 samples. Error bars represents SEM. qRT-PCR data were obtained from one to three clones each derived from three unrelated individuals. Student's *t*-test analysis was used to calculate *P* values associated with the qRT-PCR data. FC, fold change; qRT-PCR, quantitative reverse transcription-polymerase chain reaction; SEM, standard error of the mean.

Surprisingly, very few of these genes were identified in the list of D12 versus D40 cardiomyocyte DEGs, the exceptions being the acetylcholine-responsive potassium channel genes *KCNJ3* and *CACNA2D1*, encoding a subunit of the L-type calcium channel (Fig. 5A). While this potassium current is associated with atrial cardiomyocytes and has been reported to be present in human embryonic stem cell-derived cardiomyocytes, it must be activated by a muscarinic agent such as carbachol [13]. Therefore, its functional implications within our study are unclear.

Notably, though, gene function enrichment analysis of the D40 DEGs also revealed three ion channel regulator genes, *SLMAP*, *FGF12*, and *FHL1*, which are characterized by the terms “regulation of membrane depolarization” and/or “regulation of voltage-gated sodium channel activity” and thought to play a role in cardiomyocyte function [36–38]. We confirmed the increased expression of these genes in D40 versus D12 cardiomyocytes via qRT-PCR (Fig. 5B–D). The somewhat higher *P* values observed for the qRT-PCR confirmations of *SLMAP* and *FHL1* could be due to the presence of contaminating noncardiomyocytes, which were excluded in the scRNA-seq analysis.

*FHL1* has been shown to interact with the I_Kur_ channel subunit K_v_1.5 and to alter I_Kur_ functional properties when coexpressed with *KCNA5* in a heterologous expression system [38]. We therefore chose to study the role *FHL1* plays in the electrophysiology of hiPSC-CMs, and whether its increased expression with time in culture could have implications for the phenotyping of hiPSC-CMs.

### Influence of FHL1 expression on atrial-associated hiPSC-CM properties

**FIG. 6.**
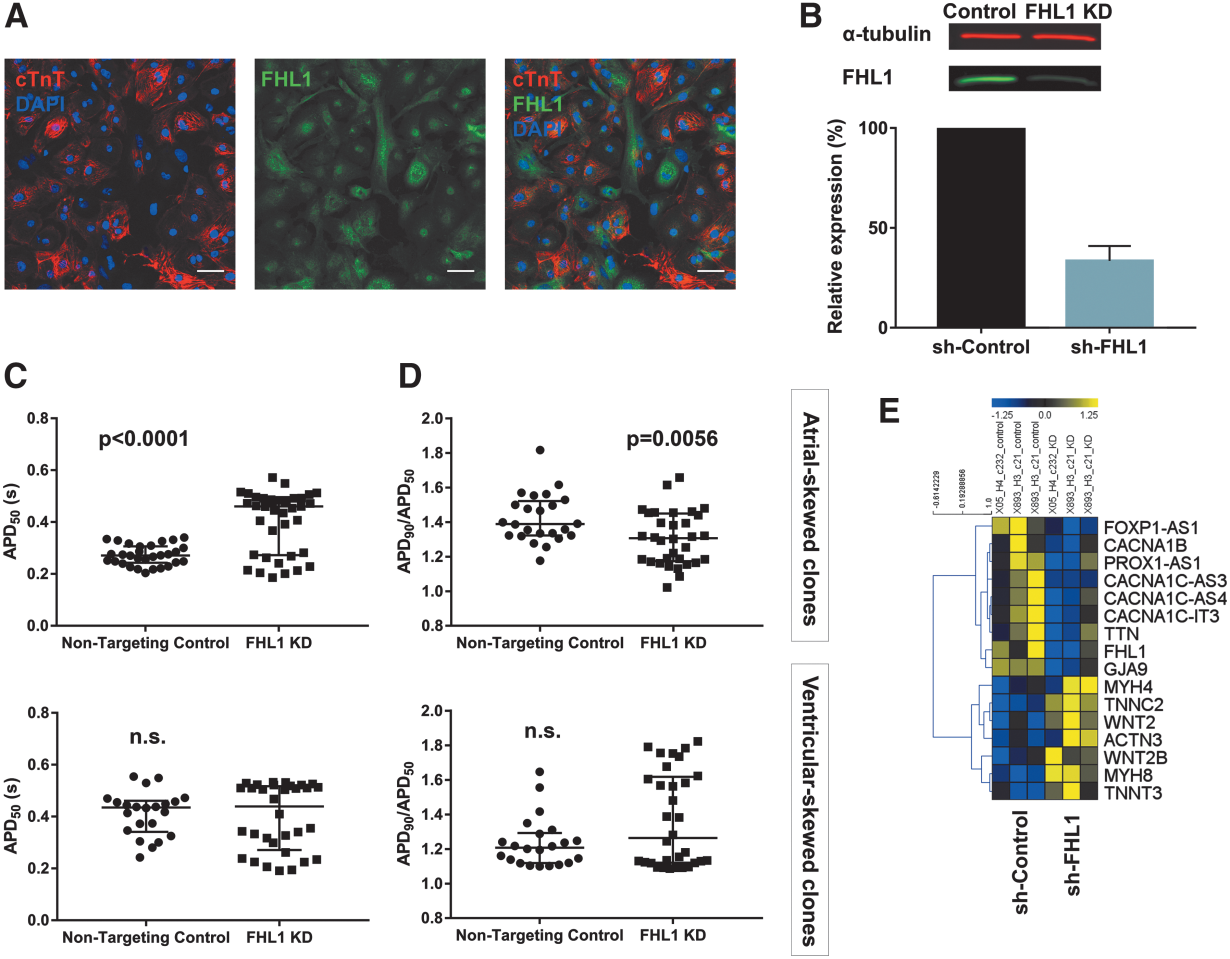
Reduced expression of *FHL1* shifts hiPSC-CM AP morphologies away from atrial-like properties. **(A)** Immunofluorescence of cTnT and FHL1 at day 40 (representative of three analyzed clones). Scale bar represents 50 μM. **(B)** Representative western blot and quantification from five clones (representing three unrelated individuals) showing *FHL1* knockdown efficiency via shRNA. Data are reported as mean ± SEM. **(C)** Cells from clones that had a mean APD_90_/APD_50_ > 1.4 in the control condition had increased APD_50_ with *FHL1* knockdown. Data were collected from same differentiations as represented in **(B)**. **(D)** Cells from clones that had a mean APD_90_/APD_50_ > 1.4 in the control condition had decreased APD_90_/APD_50_ with knockdown of *FHL1*. Data for **(C, D)** are reported as median ± the interquartile range. **(E)** Heatmap of differentially expressed genes from *FHL1* knockdown with potential involvement in cardiomyocyte development (*FOXP1-AS1*, *PROX1-AS1*, *WNT2*, *WNT2B*) or function (electrophysiology: *CACNA1B*, *CACNA1C-AS3*, *CACNA1C-AS4*, *CACNA1C-IT3*; gap junction: *GJA9*; contraction: *TTN*, *MYH4*, *TNNC2*, *ACTN3*, *MYH8*, *TNNT3*). *P* values were calculated by either a Student's *t*-test or Mann–Whitney U test. shRNA, short hairpin RNA.

Immunofluorescence staining conducted at D40 of differentiation revealed that the FHL1 protein is expressed in both cardiomyocytes (cTnT-expressing cells) and noncardiomyocytes (Fig. 6A), consistent with its expression in murine hearts [39]. To determine if it regulates AP morphologies, *FHL1* was knocked down using short hairpin RNAs (shRNAs) and compared with nontargeting control shRNA. A total of five cell lines from three unrelated individuals were analyzed. The average knockdown efficiency was 67% at the protein level (Fig. 6B). For electrophysiological analysis, an RFP reporter was used to confirm expression of either nontargeting control shRNA or *FHL1*-targeted shRNA in each cell before ArcLight imaging.

As expected, there was variation between the five cell lines in the AP properties of the control cells. Three of these had slightly more atrial-like APs (mean APD_90_/ADP_50_ > 1.4), while two were more ventricular like (mean APD_90_/APD_50_ < 1.4, along with longer APD_50_). We analyzed these two groups separately and found that the atrial-skewed cell lines were particularly responsive to knockdown of *FHL1*, demonstrating significantly longer APD_50_ and lower APD_90_/APD_50_ following the knockdown (Fig. 6C, D). Furthermore, *FHL1* knockdown promoted a shift toward larger values of AP amplitude and V_max_ in the atrial-skewed clones, also consistent with the cells adopting more ventricular-like electrophysiology (Supplementary Fig. S9) [11,13,40].

This demonstrated that expression of a single gene, *FHL1*, influences the presence of atrial-like APs from hiPSC-CMs and its variable expression during the differentiation process likely impacts cellular electrophysiology.

While members of the FHL1 family of proteins are not transcription factors, they can influence gene expression by directly interacting with transcription factors such as NFATc1 and members of the CREB family [41,42]. Therefore, we wanted to determine whether *FHL1* expression has any impact on the cardiac development process, cardiomyocyte subtype marker genes, or any other electrophysiology-related genes. To that end, we performed RNA-seq on *FHL1* knockdown cells and their corresponding controls (Supplementary Fig. S10A, B). Overall, enriched pathways for the control samples included those related to cell division, muscle filaments, signaling, and development. Enriched pathways for the knockdown samples included transport and skeletal muscle thin filament assembly (Supplementary Fig. S10C).

There was no significant change in *KCNA5* expression with *FHL1* knockdown, suggesting that altered expression in K_v_1.5, at least at a transcriptional level, is not a driving mechanism for *FHL1*'s influence on hiPSC-CM electrophysiology. We did see altered expression of genes that play a role in muscle filaments, consistent with the established role of the FHL1 protein in the contractile apparatus [43,44] (Fig. 6E). Modulation of *FHL1* additionally impacted several gap junction or calcium ion channel-related genes. Finally, we also observed differential expression of several transcripts associated with cardiac development. However, with the exception of *WNT2*, the identified genes have not been specifically implicated in cardiomyocyte subtype specification.

## Discussion

In this study, we profiled the heterogeneous nature of hiPSC-CMs, providing new insight into how gene expression and the electrophysiological features of these cells relate to each other throughout the first ∼1.5 months of the differentiation process. Thereby, we have observed numerous indications of transcriptional and electrophysiological maturation of hiPSC-CMs over the time course in this study. We also pinpointed some robust early (day 12) and later (day 40) gene markers of hiPSC cardiac differentiation based on the true single-cell signals with scRNA-seq technology. However, we were not able to identify distinct cardiomyocyte subtypes in our in vitro cell cultures at day 40 using classical markers, highlighting the challenges of using hiPSCs in vitro to mimic in vivo organogenesis.

Furthermore, we found that expression of ion channel regulator genes *SLMAP*, *FGF12*, and *FHL1* increased with time in culture, and therefore had the potential to impact the electrophysiological maturation of these cells, despite not previously being classified as cardiomyocyte subtype marker genes. Specifically, we discovered that changes in *FHL1* expression can shift electrophysiological parameters toward those that are more characteristically atrial-like or ventricular-like. Overall, our data emphasize that hiPSC electrophysiological properties, which are important for in vitro applications, are shaped by complex molecular influences in the absence of native embryology, and thus may require further dissection of noncanonical pathways that are specifically required for stem cell differentiation and bioengineered applications.

In recent years, many research groups have described individual hiPSC-CMs as “ventricular-like,” “atrial-like,” or “nodal-like” based on AP morphology. This practice had been generally accepted until Du et al. challenged the premise that these distinct electrophysiological subtypes exist in a standard cardiac differentiation [9,10]. This leads to the question of whether in vitro bioengineered CMs are capable of fully recapitulating cells isolated from in vivo systems in this manner, as in vivo cardiomyocytes are specified at very distinctive microenvironments in the early embryo and matured in an ever-changing physiological structure [10].

Therefore, we sought to examine the relationship between gene expression and electrophysiological markers of cardiomyocyte subtypes, recognizing that a more in-depth understanding of the relationship between molecular and functional properties could be broadly applicable to diverse cell types differentiated in vitro from hiPSCs.

We chose to use the genetically encoded voltage indicator ArcLight to perform the electrophysiological characterization, as this approach has the advantages of being noninvasive, nontoxic, and conducive to rapid and high-throughput electrophysiological assessments [45,46]. Importantly, data acquired via ArcLight analysis correspond well to patch clamp recordings [46]. Moreover, ArcLight permits examination of parameters of interest on a cell-by-cell basis, an important consideration given our desire to examine cell-to-cell heterogeneity within this system. ArcLight was introduced into the cells once they were already cardiomyocytes, typically within a week of analysis, thereby minimizing any potential developmental issues arising from genomic integration in our model system.

To establish a system for distinguishing subpopulations of hiPSC-CMs with atrial- and ventricular-like electrophysiology, we relied on manipulation of I_Kur_ as a gold standard functional marker, as this ion current contributes heavily toward the distinctive atrial AP morphology. Ultimately, we found that a subset of cells had AP morphologies that were sensitive to inhibition of this ion current by DPO-1, and that in general those cells had a shorter AP duration and larger APD_90_/APD_50_, as expected for atrial-like APs [11]. This confirmed that this system was sufficient to distinguish between physiologically responsive atrial-like and nonatrial-like APs.

When we examined how APD_90_/APD_50_ changed with time, we saw that cells with an atrial-like AP profile became more common after D30. It is well established that hiPSC-CMs can mature with time, and indeed, the cells in our study also demonstrated enhanced electrophysiological maturation at this later stage of differentiation by multiple measures, including both AP properties and response to tetrodotoxin [31].

A study by Burridge et al. showed via single-cell real-time RT-PCR that many D20 cardiomyocytes coexpressed markers of human atrial, ventricular, and nodal cells, suggesting that their subtype specification was not fully determined. However, immunofluorescent staining of atrial and ventricular isoforms of MLC2 exhibited a disparate expression pattern after D30 [11]. It has also been previously demonstrated that electrophysiological properties of hiPSC-CMs differentiated with a retinoic acid protocol are notably more atrial like at D40 compared with D25 [46].

Together, this led us to profile individual cells via ArcLight and scRNA-seq at early (D12) and later (D40) stages of differentiation, and thus evaluate the cells both before and after distinct cardiomyocyte subtypes might presumably be detected. Electrophysiological classification of cardiomyocyte subtypes has frequently been performed during this time frame, adding additional relevance to our study design [11,24–29].

We were able to validate numerous aspects of our scRNA-seq data in additional cell lines via qRT-PCR, immunofluorescence, and by comparison with previously published studies, thus supporting the robustness of our findings. Increased transcriptional maturation between the early and late time points in our study was also apparent from the scRNA-seq data. However, we were surprised by the degree to which classical markers of ventricular, atrial, and nodal cells did not clearly segregate even at D40. Moreover, only a few of these markers were differentially expressed between the two time points. These findings indicate that although some cells may appear more atrial like or ventricular like by electrophysiology, this does not necessarily mean they are a distinctly atrial-like or ventricular-like cell according to transcriptional profiling.

Our results suggest that there are vital roles of in vivo reciprocal cross talk between different layers of cells and tissues and the in vivo mutually reinforcing program directing native cardiac development and guiding tissue maturation, which are not fully recapitulated in vitro. Furthermore, mature cardiomyocyte subtypes have previously been delineated by scRNA-seq during mouse heart development using in vivo tissue dissection instead of an iPSC-derived cell culture system [33].

In our in vitro study, we took advantage of Fluidigm C1 system to select cells based on size, with cells <10 or >17 μm excluded from the analysis. Although we had filtered out some smaller and larger cells, we anticipated that this size-restricted strategy could overestimate the homogeneity of the selected cell population and thus generate an artificially enriched signal in hiPSC-CM. Despite this experimental bias, our results indicate a far greater heterogeneous gene expression profile within these well-characterized functional cardiomyocytes than has previously been published.

When we sought to reconcile the gene expression data with our electrophysiological findings, we identified increased expression of the gene *FHL1* at D40 within a background of minimal changes in the expression of classical electrophysiology-related genes. Yang et al. previously demonstrated that the FHL1 protein interacts with K_v_1.5 in the human atria. They subsequently showed that coexpression of *FHL1* with *KCNA5* in Chinese hamster ovary cells led to K^+^ currents that more closely resembled I_Kur_, suggesting that FHL1 plays a functional role in the I_Kur_ complex [38].

Our results further expanded on these studies to suggest that increased expression of *FHL1* temporally correlates with increased prevalence of atrial-like APs in hiPSC-CMs, and that reduced expression of *FHL1* can shift populations of hiPSC-CMs away from this more atrial-like phenotype. These findings demonstrate that the functional presence of I_Kur_ in hiPSC-CMs does not depend exclusively on expression of the atrial ion-channel gene *KCNA5*, particularly given that knockdown of *FHL1* did not alter *KCNA5* mRNA expression.

From our follow-up study examining the influence of *FHL1* knockdown on the hiPSC-CM transcriptome, there was no evidence to suggest that *FHL1* plays a notable role in the development of a global atrial versus ventricular transcriptional phenotype. Although the zebrafish homolog *fhlA* has been demonstrated to regulate heart chamber development [47], *Fhl1*^−/−^ mice exhibit normal heart development [44]. Therefore, in our system, *FHL1* likely plays a role in electrophysiology via either direct or indirect modulation of K_v_1.5 localization or activity. Due to its function as an adaptor protein, numerous hypotheses have been suggested regarding how FHL1 enacts its influence on I_Kur_ [48].

Unexpectedly, however, we also observed that *FHL1* knockdown cells displayed lower levels of the following calcium channel-related transcripts: *CACNA1B*, *CACNA1C-AS3*, *CACNA1C-AS4*, and *CACNA1C-IT3*. Potentially, this could have an effect on the plateau phase of the AP. It has also been reported that atrial-like hiPSC-CMs have lower expression of *CACNA1C*, as well as reduced peak I_Ca,L_ amplitude and current density [49]. However, the precise functional roles of the antisense and intronic transcripts we identified remain unclear.

We also identified that two other ion channel regulator genes, *SLMAP* and *FGF12*, exhibited increased expression at D40. Specific *FGF12* mutations reduce Na^+^ channel current density [37], whereas mutations in *SLMAP* are implicated in decreased membrane surface expression of hNa_v_1.5 via impaired intracellular trafficking [36]. It is possible that increased expression of one or both of these may contribute to the electrophysiological maturation of hiPSC-CMs with extended time in culture, since V_max_ and AP amplitude are heavily influenced by sodium current properties. Still, it is important to note that this study only focused on gene expression changes for these candidate genes, so further work is necessary to determine how these or other changes in ion channel assembly or trafficking could contribute to the electrophysiological phenotypes we observed.

Altogether, these findings illustrate the complex nature of in vitro hiPSC-CM electrophysiology, which is likely influenced by a wide variety of genes and environmental factors, and therefore may not fully align to the expression of molecular markers for canonical cardiomyocyte subtypes.

Although the first published scRNA-seq study of human pluripotent stem cell-derived cardiomyocytes did not report any examination of cardiomyocyte subtypes [35], a recent study also examined atrial- and ventricular-associated gene expression and electrophysiology [19].

Churko et al. uncovered distinct subpopulations of D30 hiPSC-CMs, which were enriched for *TBX5*, *NR2F2*, *HEY2*, *ISL1*, *JARID2*, or *HOPX*. They furthermore found that *HEY2* expression promoted ventricular-like electrophysiology and expression of ventricular-associated genes such as *MYL2*, whereas *NR2F2* promoted atrial-like phenotypes. These atrial-like and ventricular-like transcriptional states were more prevalent in early versus late differentiation, respectively. Notably, though, they also reported some unexpected findings, such as an inverse relationship between *NR2F2* expression and some atrial-enriched genes [19].

Interesting, we saw neither a positive or negative correlation between cellular expression of *NR2F2* and *HEY2*, nor any time-dependent changes in either of these genes. This perhaps highlights the variability of cardiac differentiations from hiPSCs, which can be further compounded by differences in cell lines, differentiation protocols, maturation states, and a variety of other cell autonomous factors. Some of our results were well correlated, for example, in our observation of higher *MYL2* and *HOPX* expression in more mature, late-differentiation hiPSC-CMs [19].

These recent scRNA-seq studies, including the data presented here, provide an excellent starting point from which to garner a better understanding of cell-to-cell heterogeneity in cardiac differentiation. Future studies will prove valuable in further deepening our understanding of all the factors that impact subtype-associated transcriptional programs and electrophysiology in hiPSC-CMs, as well as how to consistently modulate those factors in a desired manner.

Altogether, it is not entirely surprising that a model system attempting to recapitulate tissue development would face similar challenges to cardiac development inherent in the in vivo tissue environment. It must be recognized that while there are markers known to be enriched in particular cardiomyocytes subtypes, they may not be specifically expressed throughout the cardiomyocyte development, even in vivo. This may hold particularly true during the early stages of heart development [50], when cardiomyocytes exhibit a maturation state similar to that of hiPSC-CMs.

For example, expression of MLC2a is far more promiscuous during heart development [12], and other markers, including MLC2v and IRX4, are expressed at variable levels across their associated chambers (at least in the murine heart) [51,52]. Electrophysiological assessment can be hampered by similar limitations, since AP properties are variable in vivo, even in different regions of the same chamber [6,53–55]. For these reasons, the value of taking a multifaceted approach to hiPSC-CM classification has recently been highlighted [6].

While our findings have particular relevance to the derivation and application of hiPSC-CMs, we also believe they will prove an important stepping stone toward encouraging more comprehensive characterization of various types of differentiated cells derived from hiPSCs. While the easiest approach may be to simplify biology to define one marker or property as indicative of a particular cell type, in vivo systems offer far more complexity than can be accounted for by such an approach, and adult markers do not necessarily apply to developing cells with the same specificity. In the context of in vitro hiPSC-derived cells, for which many of the native developmental cues are lacking, the challenge is even greater.

Altogether, these limitations speak of the importance of ongoing efforts to enhance the maturation of hiPSC-derived cells and better recapitulate the developmental conditions of specific cell types of interest. Future single-cell analyses such as scRNA-seq will aid in demonstrating the success of such approaches to restrict cellular heterogeneity, and ultimately, single-cell analysis of human tissues will prove a valuable means of calibration. Efforts should also be focused on enhancing our ability to assess functional and molecular readouts in the same cell, building on some recent examples [14,23,56].

Overall, our data confirm the heterogeneous nature of cardiomyocyte subtype-associated marker expression and AP profiles in hiPSC-CMs. Herein, we also identified that expression of a gene (*FHL1*) previously not identified as a marker of hiPSC-CM subtypes can potentially affect electrophysiological phenotyping, whereas canonical gene expression patterns were not clearly aligned with electrophysiological properties. Therefore, our findings promote the careful consideration of multiple features to more accurately classify in vitro stem cell differentiation of cellular subtypes. Finally, our study supports a more detailed characterization of the heterogeneity of hiPSC-derived cells of other lineages, in accordance with the complex biology of in vitro developmental models required to develop the most appropriate fit-for-purpose stem cell model systems.

## Supplementary Material

Supplemental data
