# Supplementary material for: Single-Cell RNA-Sequencing and Optical Electrophysiology of Human Induced Pluripotent Stem Cell-Derived Cardiomyocytes Reveal Discordance Between Cardiac Subtype-Associated Gene Expression Patterns and Electrophysiological Phenotypes
Source: Stem Cells Dev. 2019 May 13;28(10):659–73. doi: 10.1089/scd.2019.0030 (PMC6534093; doi:10.1089/scd.2019.0030)
Supplement: Supplemental data [file Supp_Data.pdf]

Supplementary Data

Supplementary Materials and Methods

Human induced pluripotent stem cell generation, culture, and characterization

Skin biopsies were procured from deidentified healthy individuals in accordance with institutional regulations (Mayo Clinic IRB 10-006845, Clinical Trials Identifier NCT01860898). Human induced pluripotent stem cells (hiPSCs) were generated from the primary fibroblasts isolated from these biopsies by ReGen Theranostics (Rochester, MN). Sendai reprogramming and clonal selection were performed using CytoTune-iPS Sendai Reprogramming Kits (Invitrogen) according to the manufacturer's instructions. All hiPSC clones were cultured in mTeSER1 medium (STEMCELL Technologies) and split via either mechanical passaging or ReLeSR (STEMCELL Technologies).

Karyotype analysis was performed on the clone utilized for single-cell RNA-sequencing (RNA-seq) by the Cytogenetics Core at Mayo Clinic, confirming a normal karyotype. This clone and all clones used for follow-up experiments were verified to have no known damaging mutations in mitochondrial DNA (mtDNA). mtDNA sequencing was performed by the Department of Laboratory Medicine and Pathology Molecular Genetics Laboratory and the Medical Genome Facility at Mayo Clinic, and analysis of mtDNA next-generation sequencing data was done using HaploGrep software, as described previously [S1]. These same clones were also evaluated by the etoposide sensitivity assay (ESA) to confirm hiPSC quality [S2].

Cardiac differentiation of hiPSCs

hiPSCs were dispersed to a single-cell suspension using TrypLE Express (Thermo Fisher) and cultured in two monolayer stages for a total of at least 4 days before initiation of cardiac differentiation. Twenty micromolars ROCK inhibitor was used in plating. hiPSCs were differentiated to cardiomyocytes in either CDM3 medium [S3] or B-27 supplement minus insulin medium (in RPMI-1640). CDM3 medium was used for the time course and single-cell RNA-seq experiments, and follow-up validation was performed with B-27 to investigate broader generalizability of findings.

Cardiac induction was performed in confluent Geltrex® (A1413301; Thermo Fisher)-coated 96-well or 12-well plates with CHIR99021 (STEMGENT 04-0004 or S1263; Selleckchem) and 10 ng/mL of recombinant activin A (338-AC-01M/CF; R&D Systems) for 20 h. CHIR99021 concentrations were 6, 8, 10, or 12 μM, depending on what successfully produced human induced pluripotent stem cell-derived cardiomyocytes (hiPSC-CMs) for a particular clone. At day 3 postinduction, the medium was changed to fresh medium supplemented with 5 μM IWP2 (3533; Tocris) and 10 ng/mL recombinant human BMP-4 (314-BP-010; R&D Systems) for 2 days.

For experiment involving retinoic acid differentiation, medium was supplemented with 1 μm retinoic acid (Sigma-Aldrich) from day 5 through day 8. If B-27 minus insulin medium was used for differentiation, this was changed to B-27 supplement medium at day 7, or day 8 for retinoic acid differentiation. Beating cardiomyocyte cultures were subjected to lactate enrichment [S4] for 2–4 days at day 14 postinduction or after. It has previously been shown that human pluripotent stem cell-derived cardiomyocytes exhibit typical action potentials and drug responses following lactate enrichment [S4], and that cardiomyocytes enriched with more stringent glucose- and glutamine-depleted conditions show evidence of maturation from days 35 to 60 of differentiation [S5].

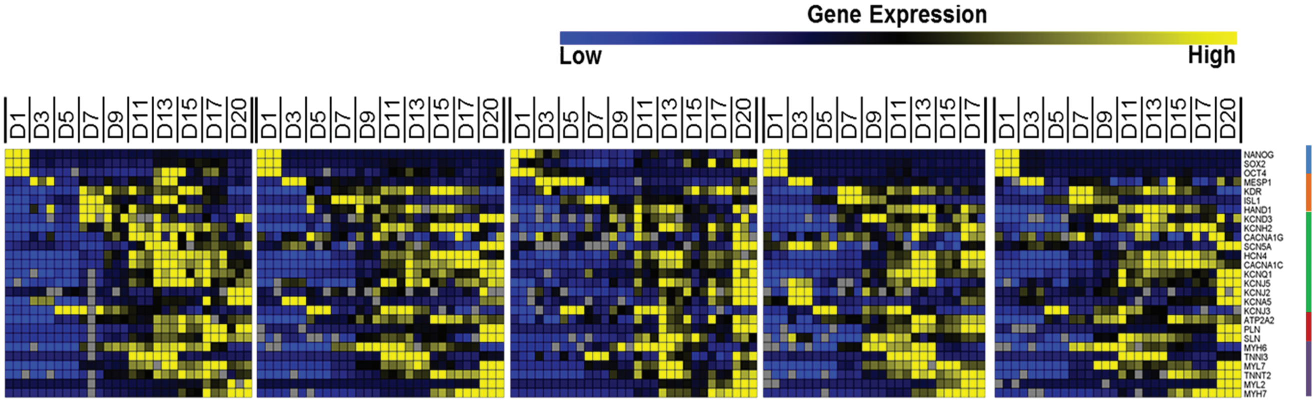

SUPPLEMENTARY FIG. S1. Shifts in population-level hiPSC-CM properties reflect cardiac differentiation and maturation. qRT-PCR analysis of cardiac differentiation from five iPSC clones of one healthy individual. RNA was collected on days 0, 1, 3, 5, 7, 9 11, 15, 17, and 20 of differentiation. Each *square* represents an individual technical replicate. *Blue* and *yellow* represent low and high expression, respectively. Missing data points are notated in *gray*. *Blue bar* on the *right* denotes pluripotency genes, *orange bar* denotes precardiac or cardiac progenitor genes, *green bar* denotes cardiomyocyte ion channel genes, *red bar* denotes cardiomyocyte calcium handling genes, and *purple bar* denotes cardiomyocyte genes involved in contractility. qRT-PCR were row-normalized to highlight differential expression patterns across the time course from days 0 to 20. hiPSC-CM, human induced pluripotent stem cell-derived cardiomyocyte; qRT-PCR, quantitative reverse transcription-polymerase chain reaction.

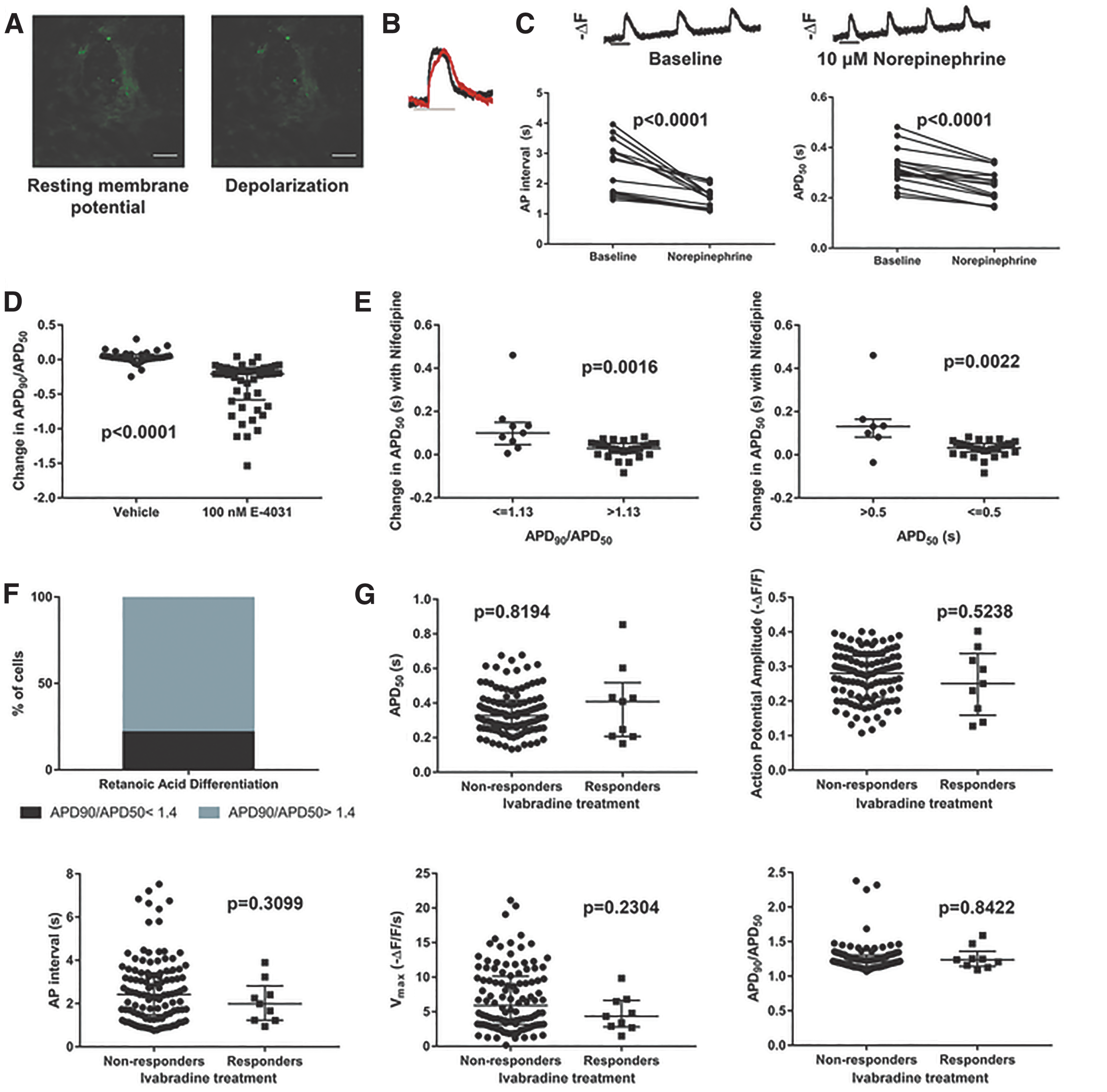

SUPPLEMENTARY FIG. S2. ArcLight allows for noninvasive electrophysiological assessment of hiPSC-CMs. **(A)** Fluorescence intensity decreases with depolarization in monolayers of ArcLight-expressing hiPSC-CMs. Scale bar represents 100 μm. **(B)** Optical APs (*black*) temporally correspond to calcium transients (*red*) reported by Rhod-3 AM. **(C)** *Top*: inverted fluorescent tracings from same cell before and after 10 μM norepinephrine. *Bottom*: change in AP interval (*left*) and duration (*right*) following norepinephrine treatment. Analysis was performed on three clones. *P* values calculated via a paired *t*-test. **(D)** hiPSC-CMs respond to treatment with hERG blocker E4031 with increased APD_{90}/APD_{50} (ratio of AP durations at 90% and 50% repolarization). ArcLight analysis was performed on three clones at day 40 of differentiation. **(E)** hiPSC-CMs with the most distinct plateaus (only those with $APD_{50} > 0.5$ s or $APD_{90}/APD_{50} < 1.13$) responded to 50 nM of L-type calcium channel blocker nifedipine with a shortened APD_{50}. ArcLight analysis was performed on three clones in nine independent experiments between days 31 and 34. **(F)** The majority (78%) of hiPSC-CMs differentiated using a retinoic acid protocol to promote atrial phenotypes were characterized as atrial like based on $APD_{90}/APD_{50} > 1.4$. Data represent four clones analyzed between days 38 and 42. **(G)** hiPSC-CM AP properties were evaluated before treatment with either 9 or 30 μM ivabradine, then parsed into responders (halted automaticity) or nonresponders. ArcLight analysis was performed on 3 clones in 10 independent experiments between days 31 and 34. All trace bars represent 500 ms. *P* values for **(D, E, G)** calculated via a Mann–Whitney U test. Data are reported as median ± interquartile range. APs, action potentials; APD_{50}, action potential duration at 50% repolarization; APD_{90}, action potential duration at 90% repolarization.

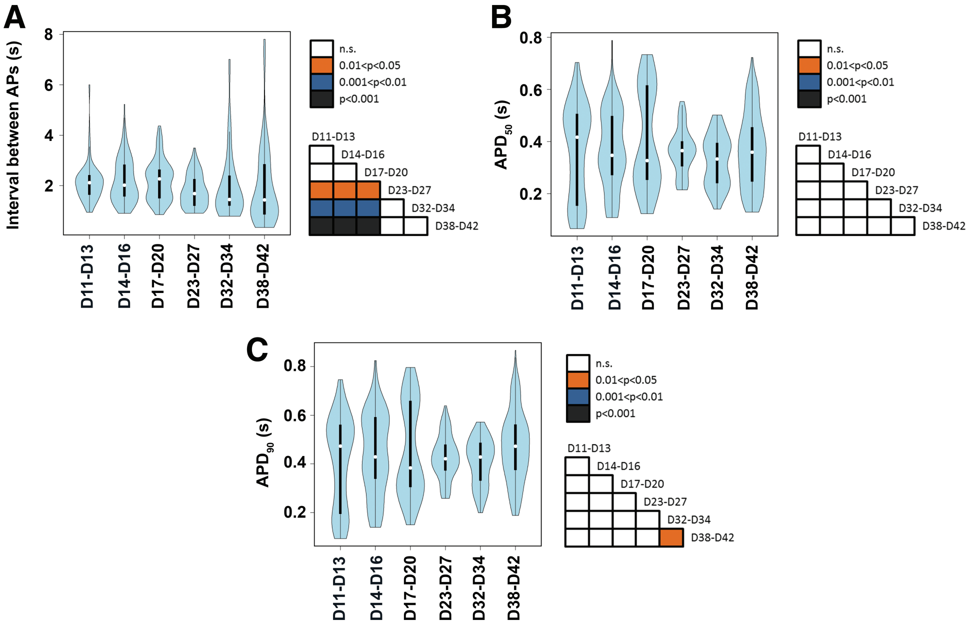

SUPPLEMENTARY FIG. S3. hiPSC-CMs demonstrate a shift in beating rate but not AP duration, with extended time in culture. **(A)** The intervals between optical APs are shifted toward shorter values after day 20 of differentiation. Eight cells for D32–D34 and six cells for D38–D42 are excluded because the interval was too long to accurately evaluate in the recording time that was used. **(B)** APD_{50} did not show any significant trend with increased time in culture. **(C)** APD_{90} values were largely unchanged with increased time in culture. Data were collected from four to seven independent differentiations per time range. Three clones from unrelated individuals are represented. Data collected from same differentiations as represented in Fig. 2A, B, and D. *White dots* within each violin indicate medians and *black rectangles* indicate interquartile range. Days 11–13 of differentiation: $n=121$ cells; D11–D14: $n=123$; D17–D20: $n=88$; D23–D27: $n=121$; D32–D34: $n=102$; D38–D42: $n=166$. Following statistical analysis via a Kruskal–Wallis test, pairwise comparisons were performed using Dunn's test with Bonferroni adjustment. Significance of pairwise comparisons is presented as *box color* in the corresponding matrix for each parameter. D, day.

Isolation of hiPSC-CMs

Single-cell hiPSC-CMs were isolated using 0.05 U of TH Research Grade Liberase (5401135001; Sigma or NATE-0994; Creative BioMart, Inc.) and 6.3 U of DNase I (D4513-1VL; Sigma) per well of a 96-well plate, followed by trypsin dissociation. hiPSCs were replated on Geltrex- or fibronectin (F1141; Sigma)-coated plates for further analysis. Nunc optical-bottomed, 96-well, black-walled plates were used for ArcLight analysis. Cells were plated to glass coverslips (NeuVitro GG-12-Pre) for patch clamp experiments.

Quantitative reverse transcription-polymerase chain reaction analysis

Total RNA was extracted with TRIzol (Invitrogen) and column purified using a Qiagen RNeasy kit. RNA was reverse transcribed to complementary DNA (cDNA) using an iScript cDNA synthesis kit (Bio-Rad). Fifteen nanograms of the cDNA was used per quantitative reverse transcription-polymerase chain reaction (qRT-PCR) in a 384-well plate. All primers were purchased from IDT (Supplementary Table S1). PCR amplification was performed using TaqMan

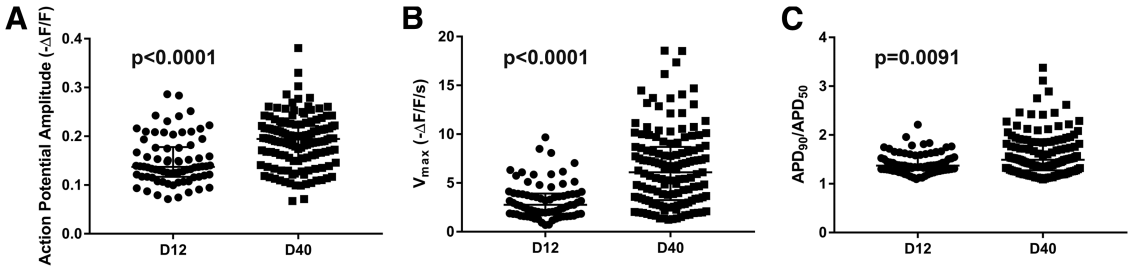

SUPPLEMENTARY FIG. S4. hiPSC-CMs differentiated with B-27 protocol demonstrate electrophysiological maturation and increased heterogeneity with extended time in culture. **(A)** Optical AP amplitudes are shifted toward larger values from D12 to D40 of differentiation. **(B)** Optical AP V_{max} is shifted toward larger values from D12 to D40 of differentiation. **(C)** APD_{90}/APD_{50} values are shifted toward larger values from D12 to D40 of differentiation. Data were collected from six clones. *P* values were calculated by a Mann–Whitney U test. Data are reported as median ± interquartile range. V_{max}, maximum upstroke velocity.

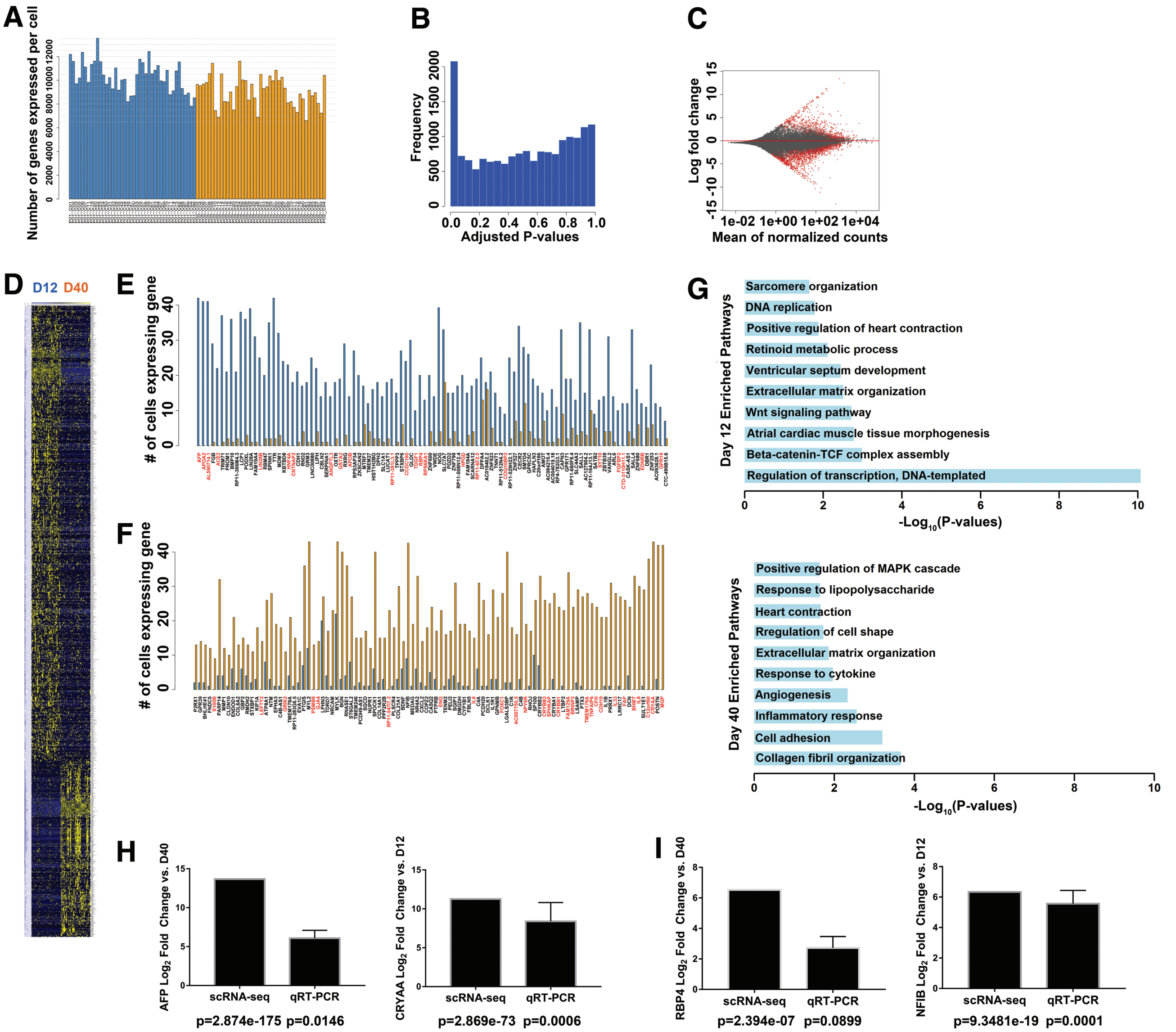

SUPPLEMENTARY FIG. S5. Cells derived from cardiac differentiation of hiPSCs exhibit transcriptional changes consistent with heart development. **(A)** Histogram representing number of genes expressed in each cell for single-cell RNA-seq experiment. **(B)** Histogram of adjusted *P* values from differential gene expression analysis of all day 12 (D12) versus day 40 (D40) cells. **(C)** Volcano plot for all D12 versus D40 cells using DESeq2. **(D)** Heatmap of DEGs between all D12 cells (*left*, 1,005 genes more highly expressed) and D40 cells (*right*, 391 genes more highly expressed). *Blue* and *yellow* represent low and high expression, respectively. **(E)** Top 100 DEGs highly expressed at D12 (*blue*) versus D40 (*orange*). Genes notated in *red* were only expressed in a maximum of one D40 cell. **(F)** Top 100 DEGs highly expressed at D40 versus D12. Genes notated in *red* were only expressed in a maximum of one D12 cell. **(G)** Enriched gene functions for the DEGs shown in **(D, H)**. Relative expression of *AFP* D12 gene expression for both scRNA-seq and qRT-PCR compared with D40, and *CRYAA* D40 gene expression compared with D12. These are genes that had the greatest difference in number of cells expressing them at D12 versus D40 in the sequencing data. **(I)** Relative expression of *RBP4* D12 gene expression for both scRNA-seq and qRT-PCR compared with D40, and *NFIB* D40 gene expression compared with D12. Both these genes are known to be involved in cardiac development. *P* values for qRT-PCR were calculated by a Student's *t*-test. DEGs, differentially expressed genes; hiPSCs, human induced pluripotent stem cells; scRNA-seq, single-cell RNA-sequencing.

universal PCR master mix (Applied Biosystems) and a ViiA7 thermocycler (Applied Biosystems). In some cases, an epMotion 5070 robotic pipettor (Eppendorf) was used to load the plate. All biological data points were generated from technical triplicates and the ΔΔCt method was used to calculate relative expression, with *GAPDH* or *ACTB* (for *FHL1* knockdown experiment) used as the reference gene.

Western blotting

To collect protein lysates, hiPSC-CMs were washed twice in ice-cold phosphate-buffered saline (PBS), then scraped off the plate, and collected in PBS. Lysis was performed for 30 min on ice using RIPA lysis buffer supplemented with Halt phosphatase inhibitor (Thermo Fisher) and complete EDTA-free miniprotease inhibitors (Roche). Protein concentration of supernatant was determined via BCA assay (Thermo Fisher).

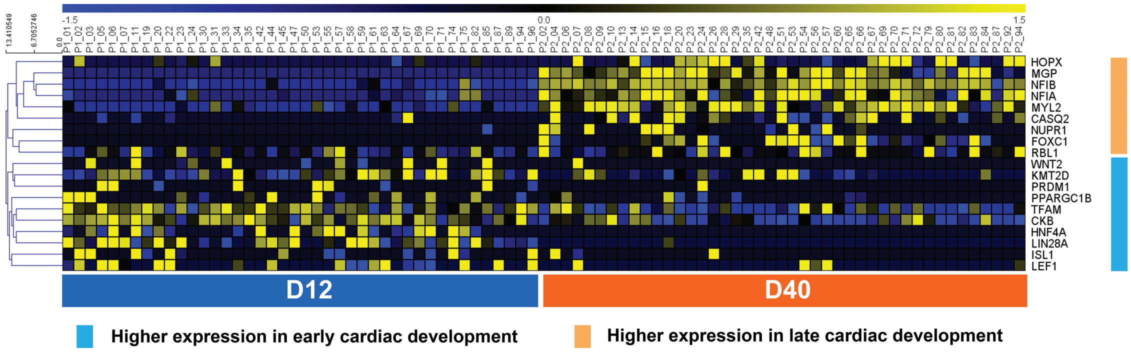

SUPPLEMENTARY FIG. S6. Cardiac differentiation of hiPSCs mirrors transcriptional changes associated with in vivo cardiac development. Heatmap of a panel of genes with consistent expression patterns between day 12 and 40 single-cell RNA-seq data (all cells) and murine heart development. All genes had $\log_2$FC >2 and *P* value of <0.05 between the two time points. FC, fold change.

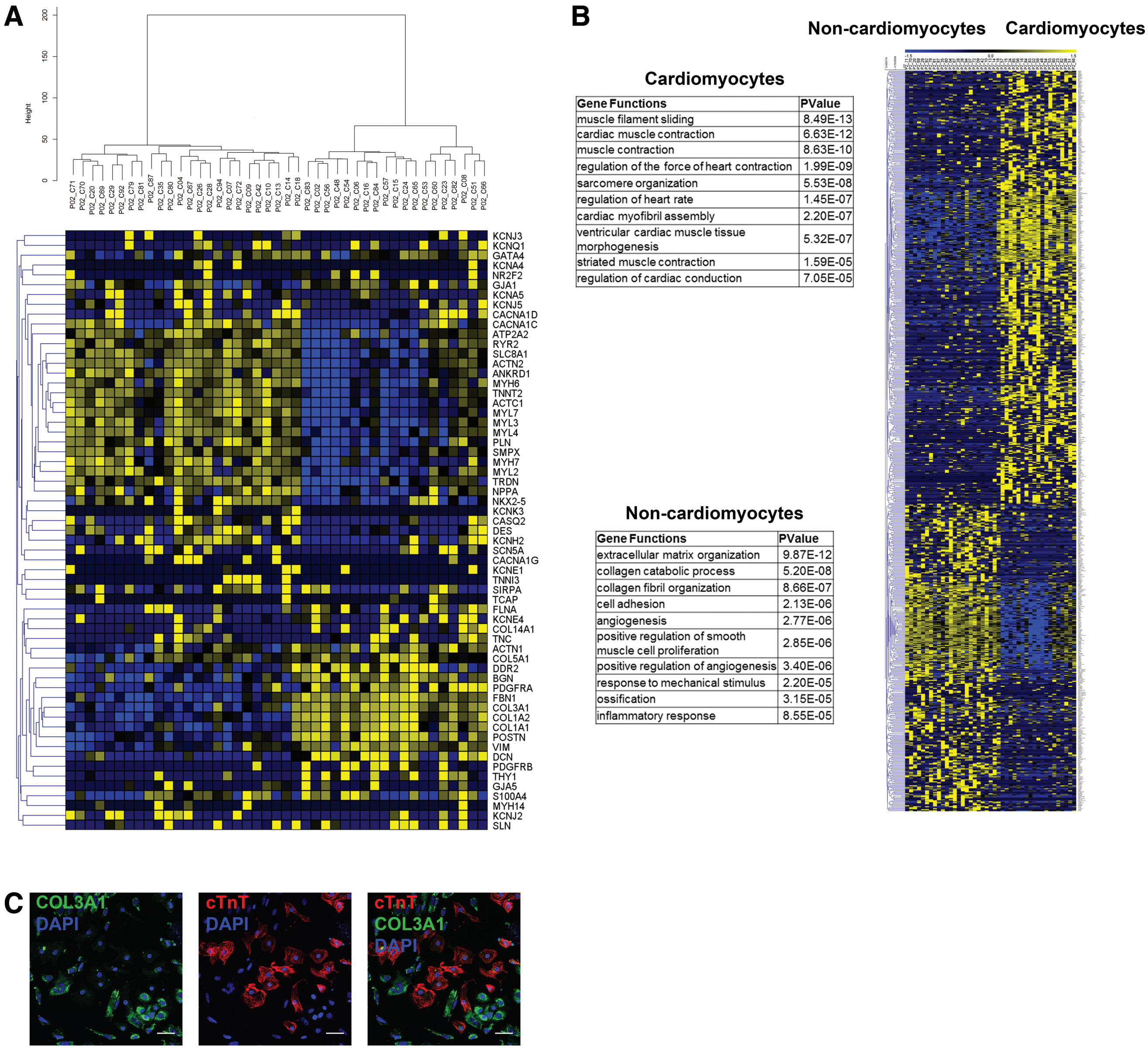

Cardiomyocytes

Gene Functions	PValue
muscle filament sliding	8.49E-13
cardiac muscle contraction	6.63E-12
muscle contraction	8.63E-10
regulation of the force of heart contraction	1.99E-09
sarcomere organization	5.53E-08
regulation of heart rate	1.45E-07
cardiac myofibril assembly	2.20E-07
ventricular cardiac muscle tissue morphogenesis	5.32E-07
striated muscle contraction	1.59E-05
regulation of cardiac conduction	7.05E-05

Non-cardiomyocytes

Gene Functions	PValue
extracellular matrix organization	9.87E-12
collagen catabolic process	5.20E-08
collagen fibril organization	8.66E-07
cell adhesion	2.13E-06
angiogenesis	2.77E-06
positive regulation of smooth muscle cell proliferation	2.85E-06
positive regulation of angiogenesis	3.40E-06
response to mechanical stimulus	2.20E-05
ossification	3.15E-05
inflammatory response	8.55E-05

SUPPLEMENTARY FIG. S7. Cardiac differentiation of hiPSCs produces heterogenous cardiomyocyte and cardiac fibroblast-like populations. **(A)** Heatmap of D40 cells clustered by expression of literature-curated cardiomyocyte and cardiac fibroblast genes. **(B)** Heatmap of DEGs between a population with higher cardiomyocyte gene expression (*right*, 310 genes more highly expressed) and a population with higher cardiac fibroblast gene expression (*left*, 434 genes more highly expressed). Top 10 gene functions are also shown. **(C)** Representative immunofluorescent image (from seven independent experiments) of expression patterns for cardiac troponin T and cardiac fibroblast marker collagen 3A1. Scale bar represents 50 μM.

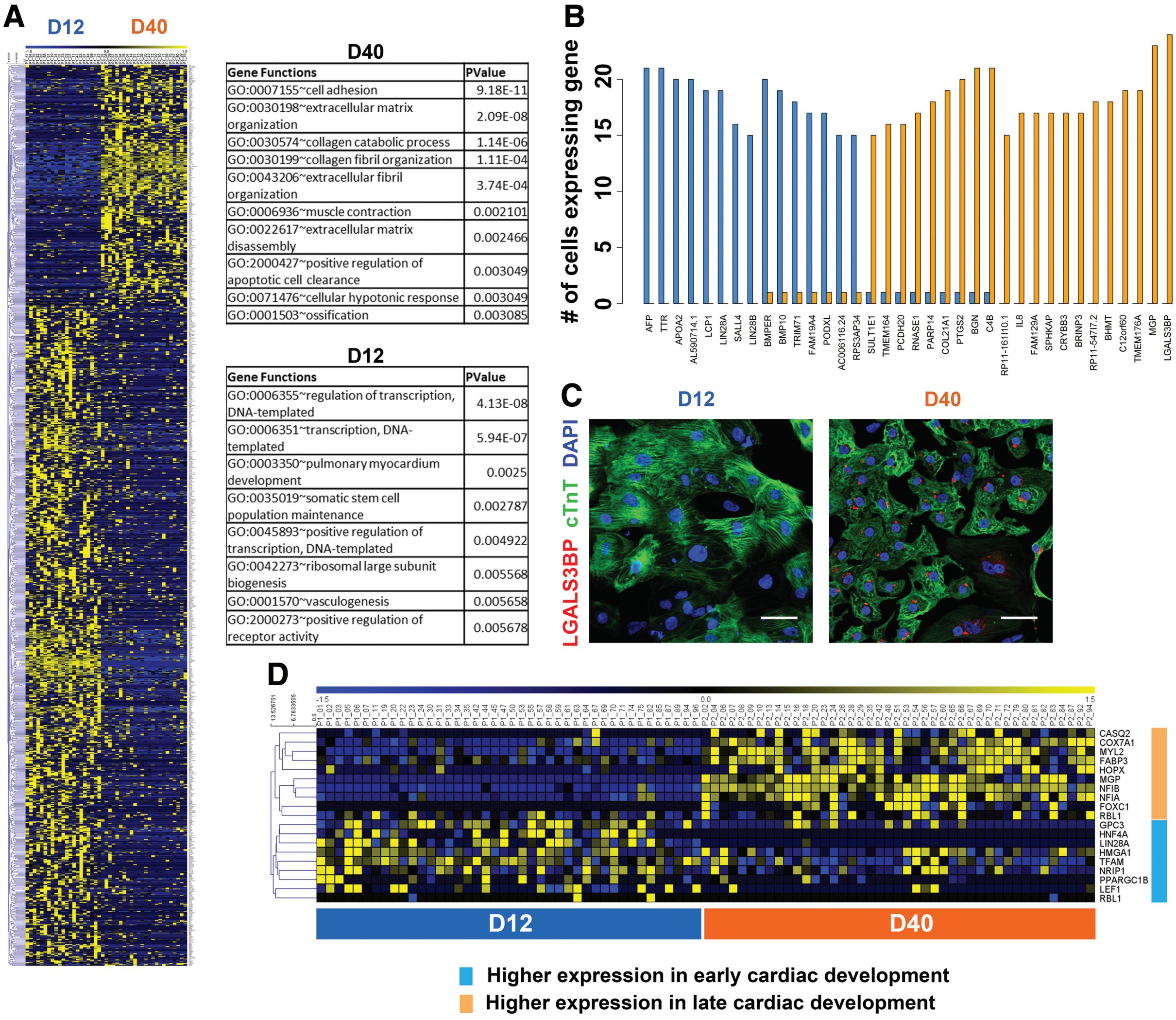

D40

Gene Functions	PValue
GO:0007155~cell adhesion	9.18E-11
GO:0030198~extracellular matrix organization	2.09E-08
GO:0030574~collagen catabolic process	1.14E-06
GO:0030199~collagen fibril organization	1.11E-04
GO:0043206~extracellular fibril organization	3.74E-04
GO:0006936~muscle contraction	0.002101
GO:0022617~extracellular matrix disassembly	0.002466
GO:2000427~positive regulation of apoptotic cell clearance	0.003049
GO:0071476~cellular hypotonic response	0.003049
GO:0001503~ossification	0.003085

D12

Gene Functions	PValue
GO:0006355~regulation of transcription, DNA-templated	4.13E-08
GO:0006351~transcription, DNA-templated	5.94E-07
GO:0003350~pulmonary myocardium development	0.0025
GO:0035019~somatic stem cell population maintenance	0.002787
GO:0045893~positive regulation of transcription, DNA-templated	0.004922
GO:0042273~ribosomal large subunit biogenesis	0.005568
GO:0001570~vasculogenesis	0.005658
GO:2000273~positive regulation of receptor activity	0.005678

SUPPLEMENTARY FIG. S8. hiPSC-CMs feature distinctive gene expression patterns at day 12 versus day 40 stages of differentiation. **(A)** Heatmap of DEGs between D12 cardiomyocytes (*left*, 732 genes more highly expressed) and D40 cardiomyocytes (*right*, 271 genes more highly expressed). Top 10 gene functions are also shown. **(B)** Biomarkers for D12 (*blue*) or day 40 cardiomyocytes (*orange*). Genes were selected based on being expressed in a maximum of one cell of the other time point. **(C)** Representative immunofluorescence image of LGALS3BP and cTnT expression at D12 and D40 (from seven independent experiments), corresponding to one of the top D40 markers from **(B)**. Scale bar represents 50 μM. **(D)** Heatmap of a panel of genes with consistent expression patterns between D12 and D40 cardiomyocytes and murine heart development. All genes had $\log_2$FC >2 and *P* value of <0.05 between the two time points.

Cell lysates were resolved on Criterion Tris-HCl 4%–15% gels (Bio-Rad). Proteins were transferred to a PVDF membrane and allowed to dry overnight. Membranes were blocked for 1 h in TBS Odyssey blocking buffer (LiCor) before incubation with a primary antibody for FHL1 at 1:250 (HPA001040; Sigma-Aldrich). An anti-α-tubulin at 1:5,000 (ab7291; Abcam) was used as a loading control. IRDye® 680LT goat anti-mouse IgG1 and IRDye 800CW goat anti-rabbit IgG secondary antibodies (LiCor) were subsequently used. The membranes were imaged and band intensities quantified using Odyssey® Imaging System and Image Studio software.

Immunocytochemistry

hiPSC-CMs were plated on Nunc Lab-Tek eight-well chamber slides (Thermo Fisher) coated with fibronectin (F1141; Sigma) and subsequently fixed in 4% paraformaldehyde for 15 min. Following fixation, they were washed three times with PBS and stored in PBS at 4°C until staining. Cells were later permeabilized in 1% Triton X-100 for 30 min, washed three times with PBS, and blocked for 3 h at room temperature in Super Block (Thermo Fisher). The cells were subsequently incubated with primary antibodies against cTnT (ms-295-P0; Thermo Fisher), COL3A1 (ab7778; Abcam), LGALS3BP (ab67353; Abcam), or FHL1 (HPA001040; Sigma-Aldrich) at dilutions of 1:200, 1:250, 1:500, or 1:50, respectively. Antibody dilution buffer was PBS containing 10% Super Block and 0.1% Tween.

Cells were incubated overnight at 4°C on a rotator and subsequently washed three times in a postantibody wash buffer of PBS plus 0.1% Tween. They were then incubated for 1 h in the dark with Alexa Fluor 568-conjugated anti-mouse IgG or Alexa Fluor 488-conjugated anti-rabbit IgG

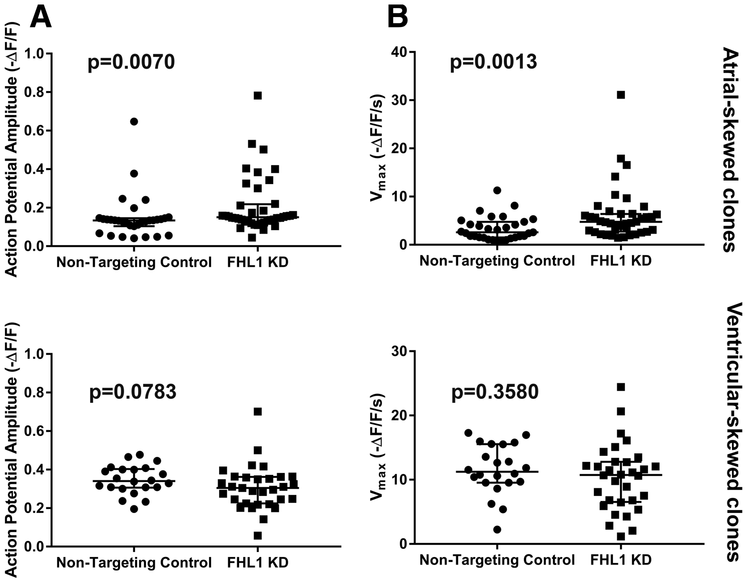

SUPPLEMENTARY FIG. S9. Reduced expression of *FHL1* modulates hiPSC-CM AP properties. **(A)** Cells from clones that had a mean $APD_{90}/APD_{50} > 1.4$ in the control condition had increased AP amplitude with *FHL1* knockdown. **(B)** Cells from clones that had a mean $APD_{90}/APD_{50} > 1.4$ in the control condition had increased V_{max} with *FHL1* knockdown. Data collected from same differentiations as represented in Fig. 6C and D. *P* values were calculated by either a Student's *t*-test or Mann–Whitney U test. Data are reported as median ± interquartile range.

secondary antibody (Invitrogen) diluted 1:500 in antibody dilution buffer. Cells were washed two times with post-antibody wash buffer, followed by two PBS washes. They were then incubated with DAPI in PBS (Invitrogen) for 8 min, washed three times with PBS, and once with distilled water. Slides were treated with ProLong Gold antifade (Invitrogen) and covered with coverglass. Stained slides were stored in the dark at 4°C until analysis using a Zeiss LSM 780 confocal microscope at 20×.

Cloning and plasmid production

The *A242-ArcLight* plasmid (plasmid 36857; Addgene), Open Biosystems LentiORF pLEX-MCS Vector (Thermo Scientific), and SMARTvector lentiviral human FHL1 or nontargeting control 1 CAG-TurboRFP short hairpin RNA (shRNA; GE Healthcare) were grown in *Escherichia coli* cells and isolated using a plasmid maxi kit (Qiagen).

The open reading frame for ArcLight was PCR amplified using KAPA HiFi HotStart PCR kit (Fisher Scientific) from the ArcLight plasmid, using the following primers to add *Not*1 (forward) and *Xho*1 (reverse) restriction sites on either side: forward: 5′-ATTATAGCGGCCGCATGGAGGGATT CGACGG-3′, reverse: 5′-GCGCCCCTCGAGTCATTTGT ATAGTTCATCCATGC-3′.

PCR products were run through an agarose gel and isolated using the QIAquick Gel Extraction Kit (Qiagen). This PCR product was cloned into a TOPO TA cloning vector. The insertion and full ArcLight insert sequence were verified by Sanger sequencing in the Mayo Clinic Medical Genome Facility using the following primers: M13 forward: 5′-GTAAAACGACGGCCAG-3′, M13 reverse: 5′-CAGGAA ACAGCTATGAC-3′; ArcLight Internal forward 1: 5′-CG GGATGGCTTTGGCTCTTTC-3′, ArcLight Internal forward 2: 5′-TTTCAAGAGTGCCATGCCCG-3′; ArcLight Internal reverse 1: 5′-CCTTCGGGCATGGCACTCTTG-3′, ArcLight Internal reverse 2: 5′-CCCGTAGGCAAATATCC TTAATCC-3′.

The ArcLight open reading frame was subsequently excised using *Not*1 and *Xho*1 (New England Biolabs). The resulting product was ligated into the LentiORF pLEX-MCS vector, transformed into DH5-alpha-competent bacteria, and the final plasmid isolated via plasmid maxi kit. The presence of the ArcLight insert in the LentiORF pLEX-MCS vector was confirmed by restriction digest and gel electrophoresis, followed by Sanger sequencing using the following primers: forward: 5′-CACCAAAATCAACGGGACTT-3′, reverse: 5′-ATATAGACAAACGCACACCGGCCT-3′.

Lentiviral production and transduction

Lentiviral vectors were packaged in HEK293 cells using the Thermo Fisher Trans-Lentiviral Packaging Kit (GE Healthcare), according to the manufacturer's instructions, and collected at 48 and 96 h after the low-serum media change. Following centrifugation to separate viral particle-containing supernatants from nonadherent cells and debris, viral particles were concentrated ∼1:50 using PEG-it Virus Precipitation Solution (System Biosciences), according to the manufacturer's instructions. Lentiviral titers were determined using a qRT-PCR-based lentiviral titer kit assay (MellGen Laboratories) and an Illumina Eco qPCR machine.

hiPSC-CMs were typically transduced with the ArcLight lentivirus at 150,000 infectious units (ifu) per well of a 96-well plate in the presence of 8 μg/mL polybrene (Millipore Sigma). For the SMARTvector shRNA lentiviruses, a total of 60,000 ifu/well was used to transduce the cells at 3 weeks of differentiation. For the *FHL1* knockdown, this comprised three pooled distinct shRNAs. shRNA transduction was followed by selection with 0.5 μM puromycin for ∼2 weeks. Transduction

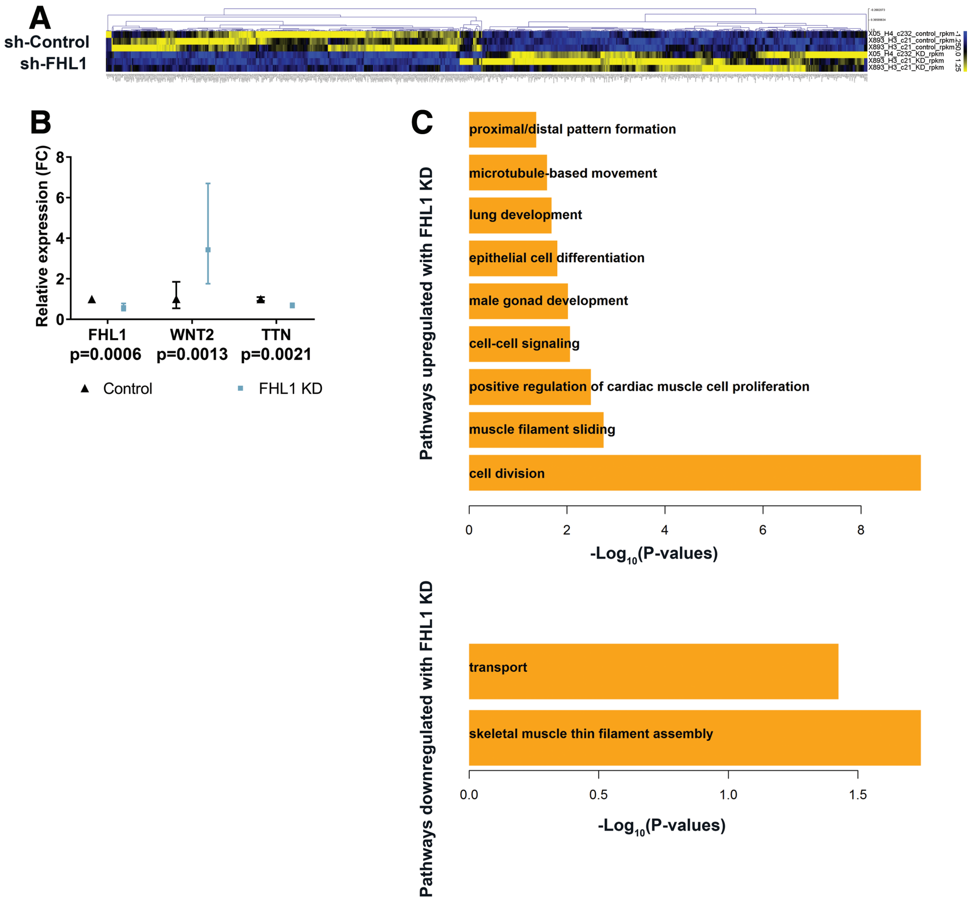

SUPPLEMENTARY FIG. S10. Influence of *FHL1* knockdown on global transcription in hiPSC-CMs. **(A)** Heatmap of DEGs after *FHL1* knockdown compared with controls (621 genes upregulated and 450 genes downregulated), with *yellow* representing higher expression and *blue* representing lower expression. **(B)** qRT-PCR validation of DEGs identified following knockdown of *FHL1*, compared with controls. Error bars represent the range of FC, as calculated from standard deviation of ΔΔCt. *P* values were calculated from ΔΔCt values by a Student's *t*-test. **(C)** Enriched gene functions of potential functional relevance.

with ArcLight lentivirus was performed after the selection process for the cells in the knockdown experiment.

ArcLight imaging and analysis

ArcLight-transduced cardiomyocytes were allowed to recover for at least 2 days after plating before ArcLight analysis was performed. Media were exchanged for Tyrode's solution (Sigma-Aldrich) before imaging. All data acquisition was performed in a live-cell incubation chamber with 5% CO_2 at 37°C. Action potentials were recorded from spontaneously beating cells using line-scan mode of a Zeiss 5 Live laser confocal microscope at 40× magnification and 500 frames per second. An FITC filter set was used for measurements of ArcLight fluorescence. For calcium imaging, cells were loaded with Rhod-3 AM calcium indicator (Thermo Fisher) before analysis, according to the manufacturer's instructions.

A custom MATLAB program (MathWorks) was written to analyze action potential parameters from the recorded data. Fluorescence intensity of the cells was corrected for background fluorescence. Following subtraction of fluorophore bleaching, optical recordings were converted to a negative change in fluorescence from baseline over fluorescence at baseline (−ΔF/F) and low-pass filtered at 50 Hz.

APD_{50} (action potential duration at 50% repolarization) was calculated as the width of the action potential at 50% its height. APD_{90} (action potential duration at 90% repolarization) was calculated as the time interval between when the signal reached 50% of maximum depolarization and when it was 90% repolarized. Maximum upstroke velocity was calculated as the maximum instantaneous slope of a curve fit to the upstroke between 10% depolarization and maximum depolarization. Action potential amplitude was calculated as the height of the action potential at its maximum depolarization. Finally, action potential interval was calculated as time between those action potential maximums.

Electrophysiological measurements and data analysis

Standard whole-cell patch clamp technique was used to measure outward currents in hiPSC-CMs at room temperature (22°C–24°C) with the use of an Axopatch 200B amplifier, Digidata 1440A, and pClamp version 10.2 software (Axon Instruments). The extracellular (bath) solution contained

(mM) 150 NaCl, 5.4 KCl, 1.8 $CaCl_2$, 1 $MgCl_2$, 15 glucose, 1 Na-pyruvate, and 15 HEPES, pH adjusted to 7.4 with NaOH. The pipette solution contained (mM) 150 KCl, 5 NaCl, 2 $CaCl_2$, 5 EGTA, 5 MgATP, 10 HEPES, pH adjusted to 7.2 with KOH [S6]. Traces elicited by depolarization of 300 ms duration to +40 mV from a holding potential of −50 mV [S7]. Action potentials from hiPSC-CMs were recorded using current clamp mode at a constant rate of 1 Hz through 5 ms depolarizing current injections of 150–300 pA [S6].

Microelectrodes were pulled on a P-97 puller (Sutter Instruments) and fire polished to a final resistance of 2–3 M′Ω. Series resistance was compensated by 80%–85%. Currents were filtered at 1 kHz and digitized at 5 kHz. Data were analyzed using Clampfit (Axon Instruments), Excel (Microsoft), and fitted with Origin 9.1 (OriginLab Corporation).

Pharmacological manipulation of ion channels

DPO-1 was purchased from Sigma-Aldrich, prepared as a stock solution in 100% dimethyl sulfoxide (DMSO), and diluted in aqueous bath solutions (for patch clamp analysis) or pertinent well (for ArcLight analysis) to achieve final concentrations of 200 nM. Norepinephrine was purchased from Tocris, prepared as a stock solution in water, and diluted to a final concentration of 10 μM. E-4031 was purchased from Abcam, prepared as a stock solution in water, and diluted to a final concentration of 100 nM. Nifedipine was purchased from Sigma-Aldrich, prepared as a stock solution in DMSO, and diluted to a final concentration of 50 nM. Tetrodotoxin was purchased from Biotium, Inc., prepared as a stock solution in water, and diluted to a final concentration of 1 or 10 μM. Ivabradine was purchased from Sigma-Aldrich, prepared as a stock solution in water, and diluted to a final concentration of 9 or 30 μM.

All aqueous test solutions had <0.1% final DMSO concentration. When ArcLight analysis was performed before and after drug treatment, cells were allowed to equilibrate for at least 15 min after the addition of the drug before any measurements were taken.

Single-cell RNA-seq and data analysis

For single-cell RNA-seq, the Fluidigm C1 system (middle-sized chip, 10–17 μm) was used to sort to individual cells from a single-cell suspension collected after ArcLight analysis. Small and large cells were discarded. Libraries were only prepared for capture sites containing a single intact cell. Paired-end sequencing was performed using an Illumina HiSeq 2500 in the Mayo Clinic Medical Genomics Facility. In total, 42 cells on day 12 and 43 cells on day 40 were sequenced.

Sequencing reads were mapped using standard messenger RNA (mRNA) sequencing workflow MAP-Rseq to generate read count matrix [S8]. Quality control was performed using RSeQC [S9]. Differential analyses were performed using DESeq R software package [S10]. Differentially expressed genes (DEGs) were selected based on P values <0.05 after false discovery rate control and log_2-fold change >2.0. Count and RPKM data, FASTQ files, and DEG analyses (day 12 vs. day 40 all cells, day 40 cardiomyocytes vs. noncardiomyocytes, and day 12 vs. day 40 cardiomyocytes) are available on request. Unsupervised hierarchical clustering analysis, principal component analysis, correlation analysis, and visualization were performed using R programming.

Functional enrichment analysis based on ''Biological Process'' gene ontology terms was performed to identify enriched pathways among DEGs. Enriched pathways on DEGs were selected by P values calculated by a Fisher test. Cells in subcluster cardiomyocyte analyses were selected based on cardiac marker expression and unbiased hierarchical clustering.

RNA-seq and data analysis

For RNA-seq of day 40 cells transduced with *FHL1*-targeted shRNA or control shRNA, paired-end sequencing was performed using an Illumina HiSeq 4000 in the Mayo Clinic Medical Genomics Facility. In total, three samples for control and three samples for knockdown were sequenced. Sequencing reads were mapped using standard mRNA sequencing workflow MAP-Rseq to generate read count matrix, and the differential analyses were performed using DESeq R software package. DEGs with P values <0.05 and log_2-fold change >1.5 were selected. Enriched pathways from the DEGs were selected according to P values calculated by a Fisher test, as done with the single-cell RNA-seq data set.

Statistical analyses

Statistical analyses for each experiment are outlined in the figure legends. All data are expressed as either mean ± standard error of the mean or median ± interquartile range, as noted in figure legends. Normality was determined using the D'Agostino-Pearson omnibus K2 test in GraphPad Prism and either a Student's *t*-test or a Mann–Whitney U test was used to for pairwise statistical comparisons. Receiver operator characteristic (ROC) curve analysis was used to determine sensitivity and specificity for DPO-1 experiment. For data presented in time course violin plots (Fig. 2), following Kruskal–Wallis test, pairwise comparisons were performed using Dunn's test with Bonferroni correction. A $P < 0.05$ was considered to be significant.

Supplementary References

S1. Perales-Clemente E, AN Cook, JM Evans, S Roellinger, F Secreto, V Emmanuele, D Oglesbee, VK Mootha, M Hirano, et al. (2016). Natural underlying mtDNA heteroplasmy as a potential source of intra-person hiPSC variability. EMBO J 35:1979–1990.

S2. Secreto FJ, X Li, AJ Smith, ES Bruinsma, E Perales-Clemente, S Oommen, G Hawse, SCL Hrstka, BK Arendt, et al. (2017). Quantification of etoposide hypersensitivity: a sensitive, functional method for assessing pluripotent stem cell quality. Stem Cells Transl Med 6:1829–1839.

S3. Burridge PW, E Matsa, P Shukla, ZC Lin, JM Churko, AD Ebert, F Lan, S Diecke, B Huber, et al. (2014). Chemically defined generation of human cardiomyocytes. Nat Methods 11:855–860.

S4. Tohyama S, F Hattori, M Sano, T Hishiki, Y Nagahata, T Matsuura, H Hashimoto, T Suzuki, H Yamashita, et al. (2013). Distinct metabolic flow enables large-scale purification of mouse and human pluripotent stem cell-derived cardiomyocytes. Cell Stem Cell 12:127–137.

S5. Tohyama S, J Fujita, T Hishiki, T Matsuura, F Hattori, R Ohno, H Kanazawa, T Seki, K Nakajima, et al. (2016). Glutamine oxidation is indispensable for survival of human pluripotent stem cells. Cell Metab 23:663–674.
S6. Ma J, L Guo, SJ Fiene, BD Anson, JA Thomson, TJ Kamp, KL Kolaja, BJ Swanson and CT January. (2011). High purity human-induced pluripotent stem cell-derived cardiomyocytes: electrophysiological properties of action potentials and ionic currents. Am J Physiol Heart Circ Physiol 301:H2006–H2017.
S7. Lagrutta A, J Wang, B Fermini and JJ Salata. (2006). Novel, potent inhibitors of human Kv1.5 K+ channels and ultrarapidly activating delayed rectifier potassium current. J Pharmacol Exp Ther 317:1054–1063.
S8. Kalari KR, AA Nair, JD Bhavsar, DR O'Brien, JI Davila, MA Bockol, J Nie, X Tang, S Baheti, et al. (2014). MAP-RSeq: Mayo analysis pipeline for RNA sequencing. BMC Bioinform 15:224.
S9. Wang L, S Wang and W Li. (2012). RSeQC: quality control of RNA-seq experiments. Bioinformatics 28:2184–2185.
S10. Anders S and W Huber. (2010). Differential expression analysis for sequence count data. Genome Biol 11:R106.
